# Mechanism-Based Screen for G1/S Checkpoint Activators Identifies a Selective Activator of EIF2AK3/PERK Signalling

**DOI:** 10.1371/journal.pone.0028568

**Published:** 2012-01-12

**Authors:** Simon R. Stockwell, Georgina Platt, S. Elaine Barrie, Georgia Zoumpoulidou, Robert H. te Poele, G. Wynne Aherne, Stuart C. Wilson, Peter Sheldrake, Edward McDonald, Mathilde Venet, Christelle Soudy, Frédéric Elustondo, Laurent Rigoreau, Julian Blagg, Paul Workman, Michelle D. Garrett, Sibylle Mittnacht

**Affiliations:** 1 Research Department of Cancer Biology, UCL Cancer Institute, London, United Kingdom; 2 Division of Cancer Biology, The Institute of Cancer Research, London, United Kingdom; 3 Division of Cancer Therapeutics, The Institute of Cancer Research, Haddow Laboratories, Sutton, United Kingdom; 4 Cancer Research Technology Discovery Laboratories London, Wolfson Institute for Biomedical Research, London, United Kingdom; National Cancer Institute, United States of America

## Abstract

Human cancers often contain genetic alterations that disable G1/S checkpoint control and loss of this checkpoint is thought to critically contribute to cancer generation by permitting inappropriate proliferation and distorting fate-driven cell cycle exit. The identification of cell permeable small molecules that activate the G1/S checkpoint may therefore represent a broadly applicable and clinically effective strategy for the treatment of cancer. Here we describe the identification of several novel small molecules that trigger G1/S checkpoint activation and characterise the mechanism of action for one, CCT020312, in detail. Transcriptional profiling by cDNA microarray combined with reverse genetics revealed phosphorylation of the eukaryotic initiation factor 2-alpha (EIF2A) through the eukaryotic translation initiation factor 2-alpha kinase 3 (EIF2AK3/PERK) as the mechanism of action of this compound. While EIF2AK3/PERK activation classically follows endoplasmic reticulum (ER) stress signalling that sets off a range of different cellular responses, CCT020312 does not trigger these other cellular responses but instead selectively elicits EIF2AK3/PERK signalling. Phosphorylation of EIF2A by EIF2A kinases is a known means to block protein translation and hence restriction point transit in G1, but further supports apoptosis in specific contexts. Significantly, EIF2AK3/PERK signalling has previously been linked to the resistance of cancer cells to multiple anticancer chemotherapeutic agents, including drugs that target the ubiquitin/proteasome pathway and taxanes. Consistent with such findings CCT020312 sensitizes cancer cells with defective taxane-induced EIF2A phosphorylation to paclitaxel treatment. Our work therefore identifies CCT020312 as a novel small molecule chemical tool for the selective activation of EIF2A-mediated translation control with utility for proof-of-concept applications in EIF2A-centered therapeutic approaches, and as a chemical starting point for pathway selective agent development. We demonstrate that consistent with its mode of action CCT020312 is capable of delivering potent, and EIF2AK3 selective, proliferation control and can act as a sensitizer to chemotherapy-associated stresses as elicited by taxanes.

## Introduction

G1/S checkpoint activation is recognized to play an important role in tumour suppression [1]. The retinoblastoma tumour suppressor protein (pRB) is a critical component in this checkpoint, acting to inhibit the transcription of genes required for DNA synthesis [2]. In addition, pRB prevents the degradation of the cyclin dependent kinase inhibitors p21^CIP^ and p27^KIP1^ by the Skp-Cullin-F-box protein (SCF) ubiquitin ligase complex [3]. Phosphorylation of pRB by the cyclin dependent kinases (CDK) 4 or 6 and CDK2 inhibits these different activities of pRB, permitting transit of cells into S-phase and facilitating DNA replication [4], [5].

G1/S checkpoint control is impaired in the majority of cancers [6], [7]. Loss of control is caused by genetic alterations that affect the functioning or expression of proteins that regulate the action of pRB. Such alterations comprise inactivating mutations or gene loss of the p16^INK4A^ CDK inhibitor, which inhibits the kinase activity of CDK4 and 6 [8]; mutations in CDK4 or CDK6, rendering these kinase catalytic subunits resistant to the action of INK4 family CDK inhibitors [9]; and the deregulated expression of D cyclin genes, arising from either gene translocation [10], [11] or, more frequently, gene transcriptional activation as a consequence of oncogene activation. Signalling through the Ras, wingless (Wnt) and nuclear factor kappa B (NFκB) pathways all result in the transcriptional activation of D cyclin genes and mutational activation of these pathways in cancers is thought to contribute to unlicensed G1/S checkpoint transit [12], [13].

Small molecule chemical probes represent important tools for understanding cell pathways and validating potential therapeutic approaches [14]. Identification of cell-permeable small molecules that trigger the G1/S checkpoint through activation of pRB may provide a promising avenue for reinstating proliferation control in the clinical control of malignant disease.

Current efforts have primarily focussed on the catalytic inhibition of the G1/S pRB-phosphorylating cyclin dependent kinases CDK4 and CDK2 [15], [16]. However, a considerable level of functional redundancy appears to exist amongst members of this kinase family, and a full complement of G1/S regulatory cyclin-CDK complexes may be essential in some but not other cell types [17], [18], suggesting that effective checkpoint activation through inhibition of these enzymes could be more problematic than anticipated.

To identify agents and alternative targets capable of delivering G1/S checkpoint activation, we undertook a mechanism-based screen, scoring for the inhibition of pRB phosphorylation in human cancer cells exposed to a library of small molecules. Here we report identification and characterization of small molecule agents that potently inhibit pRB phosphorylation and proliferation, amongst them a known kinase inhibitor with activity against CDKs. We characterize in detail one compound, CCT020312, which stood out using combined assessment of chemical properties and biological effects. This compound does not inhibit CDKs but leads to rapid loss of D cyclin expression, which we demonstrate requires signalling through the eukaryotic initiation factor 2A kinase 3 (EIF2AK3/PERK). It is known that EIF2AK3/PERK activation is caused by agents that disable ER functioning, leading to accumulation of unfolded proteins [19]. In contrast, we show that CCT020312 does not elicit a generalized unfolded protein response signal, but instead operates by a mechanism of action that selectively boosts EIF2AK3/PERK signalling output.

## Results

### Identification of compounds leading to block of pRB phosphorylation in cells

We used our previously described high-throughput assay to identify agents capable of attenuating pRB phosphorylation in proliferating HT29 human colon carcinoma cells [20], and material and methods. HT29 cells were chosen for the screen because they possess functional pRB but are deficient for the tumour suppressor TP53 [21]. This eliminates identification of undesired hits that act through TP53-mediated induction of the CDK inhibitor p21^CIP1^ and associated block of pRB phosphorylation, which is common as a response to non-specific chemical stress including chemically induced DNA damage. Hits in the assay cause loss of Ser608-phosphorylation on human pRB (P-S608-pRB), a known target of cyclin D-activated CDK4/6 [22]. Using this assay we screened the Cancer Therapeutics Unit compound library of 63,000 chemically diverse compounds by applying these at a concentration of 10 µM for 24 hours. A total of 53 compounds decreased the phospho-Ser608 pRB signal in HT29 cells by greater than 50% compared to vehicle-treated control, giving a screen hit rate of 0.08%. Eight of these hits were reconfirmed as inhibitors of Ser608 phosphorylation of pRB by western blot analysis (not shown).

**Figure 1:**
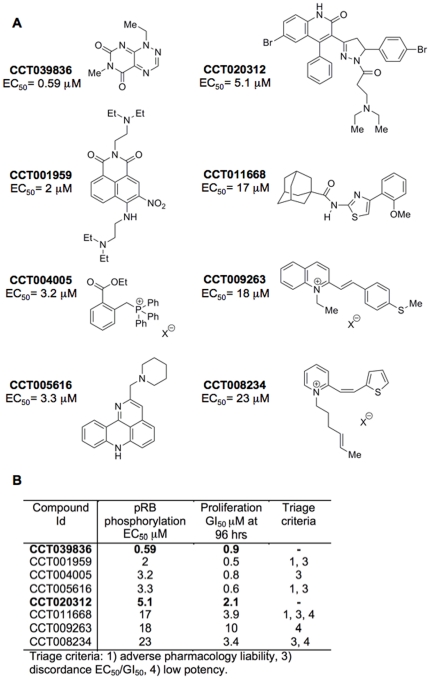
Chemical structure and properties of screen identified hits. **A) Compound chemical structures.** Compounds are arranged in order of decreasing potency. **B) Summary of cellular effects and rationale for compound triage.** EC_50_ values for inhibition of pRB phosphorylation and GI_50_ values for inhibition of cell growth represent the calculated mean (n = 3). The cell-based immunoassay for the detection of pRB-P-Ser608 (Barrie et al. 2003) was run in HT29 colon carcinoma cells and used to quantify the EC50 for inhibition of pRB phosphorylation at 24 hours, with sulphorhodamine B staining to quantify the GI_50_ values at 96 hours post compound addition. Protein remaining in wells at 24 hours was determined using bicinchoninic acid assays.

Figure 1A shows the chemical structures of the eight confirmed hits, arranged according to their potency for reducing the P-S608-pRB signal. To further assess these eight hits we considered their chemical properties (Figure 1B and Results S1) as well as biology-led criteria (Figure 1B), including good correlation between inhibition of pRB phosphorylation and growth inhibition, suggesting that the latter is a consequence of the former. These criteria favoured CCT039836 and CCT020312 (see Figure 1B) and hence these were chosen for further characterization.

A search for prior art identified CCT039836 as a CDK inhibitor [23] and our subsequent assessment showed half-maximal inhibition of recombinant CDK4/cyclin D1 enzyme at a concentration of 0.2 µM, with comparable potency against recombinant G2/M kinase CDK1/cyclin B, (not shown). Compounds with CDK inhibitory activity would be expected as hits from a cell based screen using inhibition of pRB phosphorylation as an endpoint, and identification of the known CDK inhibitor CCT039836 provided assurance of assay performance.

In contrast, addition of CCT020312 to kinase reactions containing CDK4/cyclin D or CDK1/cyclin B showed no effect on the activity of these kinases at concentrations 10 times the EC_50_ for suppression of pRB phosphorylation in cellular assays (Figure S1), nor did we find an effect on cyclin E- or cyclin A-activated CDK2 (Figure S1). Inhibition of these kinases was seen in parallel reactions containing the CDK inhibitors flavopiridol or R-roscovitine (Figure S1). Therefore we concluded that CCT020312 does not act through direct CDK inhibition.

### Cellular effects of CCT020312

**Figure 2:**
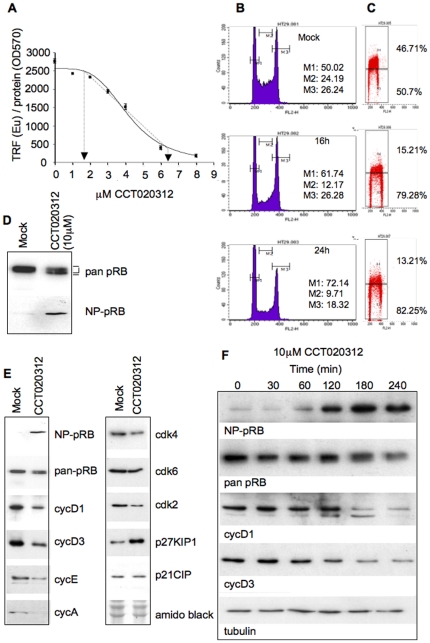
Cellular responses to CCT020312 treatment. **A) Concentration-dependence of P-S608-pRB loss in CCT020312-exposed cells.** HT29 cells seeded in 96-well plates were exposed to CCT020312 for 24 hours. Ser608 pRB phosphorylation was quantified using the cell-based immunoassay for the detection of pRB-P-Ser608 as employed for the primary screen (Barrie et al. 2003). Signals normalized to protein content (BCA assay) are shown. Error bars represent the standard error of the mean (n−3). The range for linear response is indicated. **B) C) Effects of CCT020312 on cell cycle progression and DNA synthesis.** HT29 cells were treated for 16 and 24 hours with 10 µM CCT020312. Cells were stained with propidium iodide and analysed by flow-cytometry (B). Cells were treated with CCT020312 for 16 or 24 hours. BrdU was added to the medium for the final two hours. Cells were stained with anti-BrdU antibody and analysed by flow-cytometry (C). **D) Accumulation of Ser608 unphosphorylated pRB in CCT020312 exposed cells.** HT29 cells were incubated in the presence of the vehicle (MOCK) or CCT020312 for 24 hours and analysed by immunoblotting. NP-pRB denotes use of the antibody for detection of the non-phosphorylated Ser608 pRB site. **E) Marker expression 24 h post CCT020312 exposure.** HT29 cells were exposed to 10 µM CCT020312 or vehicle (MOCK) for 24 hours. Lysates were analysed by immunoblot for marker proteins as indicated. Membrane staining with amido black documents loading. **F) CCT020312 induces a rapid loss of D cyclin expression.** HT29 cells were treated with 10 µM CCT020312 for the times indicated and lysates analyzed as in E. Tubulin probing documents loading.

Treatment of HT29 cells with CCT020312 for 24 hours revealed a concentration-dependent loss of P-S608-pRB signal, with a linear response between 1.8 and 6.1 µM (Figure 2A). Parallel assays in HCT116 colon carcinoma cells, revealed inhibition of pRB phosphorylation at comparable concentrations with half-maximal reduction of pRB phosphorylation at 4.2 and 5.7 µM in HT29 and HCT116 cells respectively (Figure S2). These concentrations closely match those required for half-maximal inhibition of growth (GI_50_) in these cell lines (GI_50_ = 3.2 and 5.4 µM respectively) (Figure S2), in line with our previous results obtained for the purpose of compound triage (Figure 1B). Lastly, CCT020312 treatment effectively inhibited cell proliferation (as measured at 96 hours) even if treatment was for 2 hours only with subsequent compound washout, indicating that CCT020312 is capable of eliciting durable rather than transient cytostasis (Figure S2).

Flow-cytometry of HT29 cells exposed to 10 µM CCT020312 (Figure 2B) revealed an increased number of cells in the G1 phase of the cell cycle at 16 and 24 hours as well as effective reduction of DNA synthesis (Figure 2C), in agreement with the expectation that loss of pRB phosphorylation results in blocking S phase entry. Immunoblotting of cell lysates confirmed the presence of fast migrating pRB in such cells and accumulation of a pRB species specifically recognised by an antibody for the underphosphorylated active form of pRB, NP-pRB [22] (Figure 2D), indicating that genuine activation of pRB signalling arises in CCT020312-treated cancer cells.

To begin to uncover the molecular mechanism responsible for the observed activation of pRB signalling and G1 arrest, we examined the effect of CCT020312 on the expression of cell cycle regulating proteins. Treatment of HT29 cells with 10 µM CCT020312 for 24 hours reduced the amount of the G1/S cyclins D1, D2, E and A as well as the CDK catalytic subunit CDK2 and increased the level of the CDK inhibitor p27^KIP1^ present in such cells (Figure 2E). The loss of D-type cyclins D1 and D3 was readily detectable 120 min after treatment (Figure 2F), coincident with the accumulation of underphosphorylated pRB, indicating that cyclin D loss could be responsible for the suppression of pRB phosphorylation. In contrast, loss of cyclin A and E and increased p27/KIP1 occurred later (Figure S3), and thus may be consequence, rather than cause, of the inhibition of pRB phosphorylation and G1/S transit. Cyclin D1 loss following CCT020312 treatment was observed across a range of different human cancer cell lines (Figure S3), including those known to express aberrantly high amounts of this cyclin.

To determine the reason for the observed cyclin D1 loss we probed for phosphorylation on Thr286 (Figure S4), which is known to target this cyclin for regulated degradation by the proteasome [24], [25] and measured the protein half-life (Figure S4). We found that neither was affected by CCT020312, nor were there detectable alterations in the cyclin D1 mRNA within the relevant time period (Figure S4), indicating that CCT020312 does not act by reducing cyclin D1 gene transcription or protein stability.

### cDNA microarray-based transcriptional profiling reveals a candidate mechanism of action

As an unbiased approach to determine the mechanism by which CCT020312 elicits its effects, we performed cDNA microarray-based transcriptional profiling using mRNA from HT29 cells treated for different times with either 10 µM CCT020312 or vehicle (Dimethyl sulfoxide, DMSO). To interpret the CCT020312-induced gene expression changes we employed Connectivity Map (cmap), a tool based on pattern matching for the discovery of functional connections between pharmacological agents [26]. This revealed close similarity to the gene regulatory signature of thioridazine, a neuroleptic dopamine antagonist without known links to cyclin D expression, and also to 15-delta prostaglandin J2, which is known to cause a rapid reduction of cyclin D1 protein through the activation of the eukaryotic translation factor 2A (EIF2A) Serine 51 (Ser51) kinase EIF2AK3/PERK, leading to inhibition of cyclin D1 mRNA translation [27], see Table S1. We note that there is no structural resemblance between CCT020312 and either of these agents, suggesting that their common transcriptional effects are based on independent chemistry.

**Figure 3:**
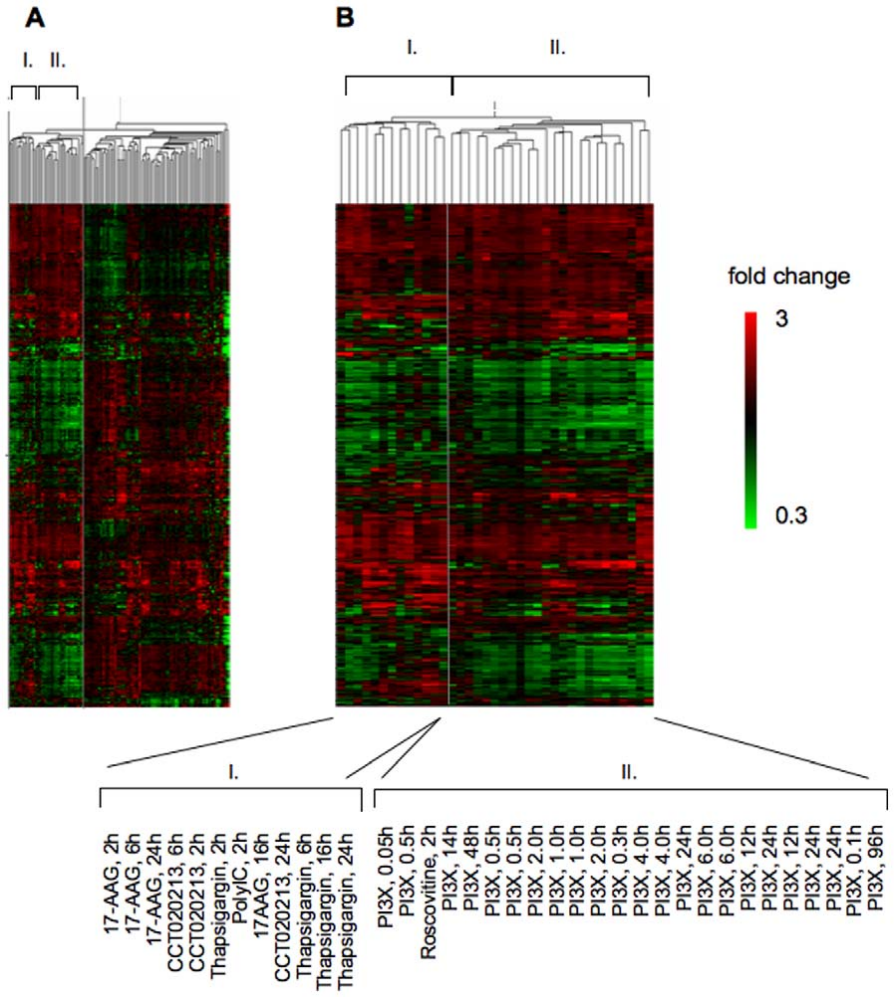
Mechanism of action predictions using cDNA microarray-based cluster analysis. **A) Cluster analysis.** Data from CCT020312 and 22 other molecularly targeted agents, 296 samples in total, established using either the CRUK Human Whole Genome-wide Array v1.0.0 or the CRUKDMF_22K_v1.0.0 array were used. Genes that significantly varied with treatment (ANOVA p<0.05 with Bonferoni-correction) were used for hierarchical clustering. Clustering was performed using the Euclidian distance as a similarity measure. **B) Cluster deconvolution.** Conditions that cluster with (Cluster I) or adjacent to (Cluster II) CCT020312 are shown.

We also performed Euclidian distance metric clustering of the CCT020312 data alongside cDNA microarray data for 22 different targeted agents previously analyzed in the Cancer Research UK Cancer Therapeutics Unit using the CRUKDMF_22K_v1.0.0 (GEO accession GPL4348) or the CRUKDMF_WGA_v1.0.0 (GEO accession GPL3904) cDNA microarray platform (Figure 3). This revealed co-clustering of CCT020312 with the heat shock protein 90 (HSP90) molecular chaperone inhibitor 17-allylamino-17-demethoxy-geldanamycin (17-AAG) and the sarco/endoplasmic reticulum Ca^2+^ ATPase targeting agent thapsigargin (Figure 3B) which, like 15-delta prostaglandinJ2, triggers EIF2A phosphorylation though EIF2AK3/PERK. A subsequently generated cDNA microarray data set from cells treated with polyinosinic-polycytidylic acid (poly (I∶C)), another effector of EIF2A phosphorylation, also clustered with CCT020312. An adjacent cluster (cluster II) contained phosphatidylinositol 3-kinase (PI3K) targeting agents with differing scaffolds (Figure 3B).

### CCT020312 elicits EIF2A phosphorylation in cells

**Figure 4:**
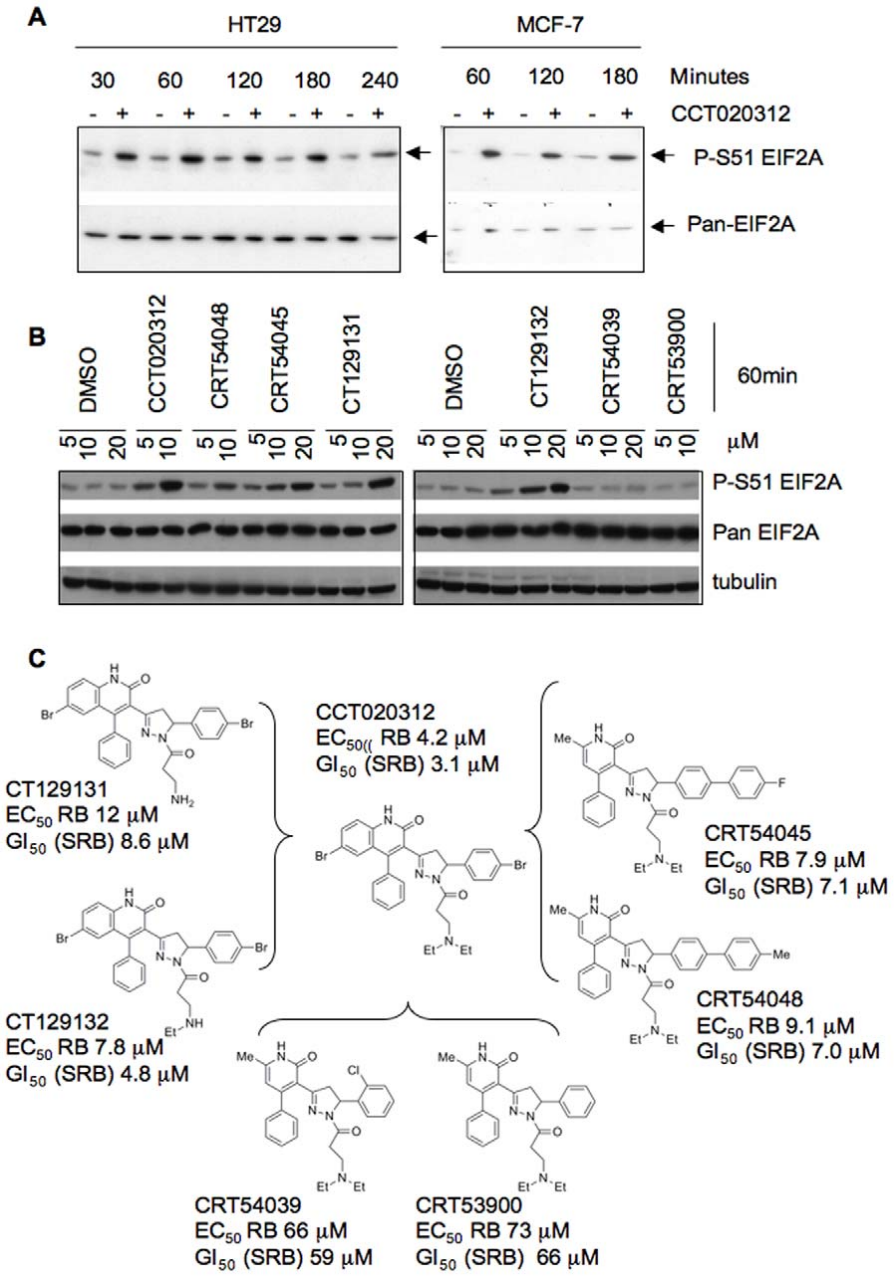
EIF2A phosphorylation in CCT020312-treated cells. **A) Detection of EIF2A phosphorylation following CCT020312 treatment.** HT29 human colon cancer and MCF-7 human breast cancer cells were treated with 10 µM CCT020312 (+) or vehicle () for the times indicated. Lysates were prepared and immunoblot-analysis performed using antibodies to detect EIF2A and Ser51-phosphorylated EIF2A (P-S51EIF2A) as indicated. **B) Structure-activity relationships.** Representatives from different chemical series were tested using HT29 cells for their respective ability to activate EIF2A phosphorylation. **C) Analogue structures and potency of analogues for reducing P-S608-pRB and growth.**

On the basis of the above mRNA profiling analysis we hypothesised that CCT020312 might act by inhibiting HSP90 or PI3 kinase or by affecting EIF2A phosphorylation. We thus probed lysates prepared from HT29 cells treated with CCT020312 for biomarkers indicative of these respective events. We found no evidence of reduced phosphorylation of the PI3K target AKT/PKB (Ser473) nor of ribosomal protein S6 kinase RPS6KB1 (Thr299), [28] (not shown), nor induction of heat shock protein 70, known to ensue from HSP90 inhibition [29] (not shown). In contrast a clear increase in phosphorylation of EIF2A on Ser51 was seen upon treatment of either HT29 or MCF7 cells with CCT020312 (Figure 4A, see Figure S5 for quantification). Rapid loss of D cyclins is a known event linked to attenuation of protein translation following EIF2A phosphorylation [30], [31] and hence readily explained by this mode of signalling. Together these results strongly support a mechanism in which CCT020312 acts as an effector of Ser51 EIF2A phosphorylation.

To determine the structural requirements for activity and biomarker modulation seen in CCT020312-treated cells we studied several analogues (Figure 4C). These studies revealed a tight correlation between growth inhibition, retinoblastoma protein activation and the activation of EIF2A phosphorylation (Figure 4B). Removal of lipophilicity from the core scaffold of CT020312 (exemplified by analogues CRT53900 and CRT54039) (Figure 4B) resulted in a substantial (16–17 fold) loss of potency in both RB phosphorylation and cell growth assays. Likewise these analogues lost the capacity to activate EIF2A phosphorylation. Analogues CRT54048 and CRT54045, in which lipophilicity was restored by the introduction of a biphenyl ring system, regained activity. Significantly these compounds also regained the ability to trigger EIF2A phosphorylation although a two-fold higher concentration was required to achieve a level of response comparable to CCT020312, consistent with the slightly reduced potency of these analogues in other assays. Analogues CT129131 and CT129132 prepared through successive removal of the two N-ethyl groups retained the ability to activate pRB and inhibit growth with only slight (two- to three-fold) reduction in potency; the ability to trigger EIF2A phosphorylation was retained although a two-fold higher concentration was again required. Hence a close structure-activity relationship exists between the inhibition of pRB phosphorylation, growth inhibition and the activation of EIF2A phosphorylation, supporting the view that these activities are mechanistically linked.

### EIF2A phosphorylation and cyclin D1 loss by CCT020312 requires EIF2AK3/PERK

**Figure 5:**
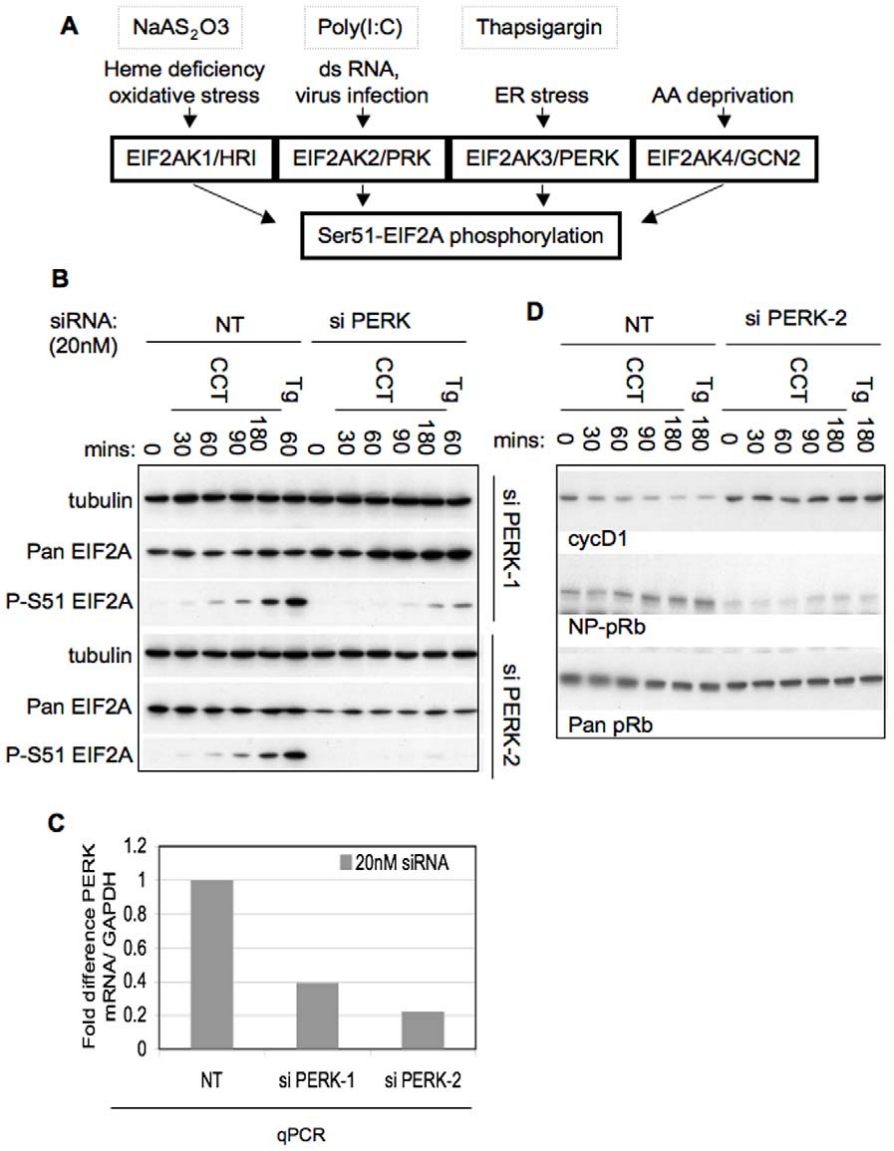
Signalling leading to CCT020312-dependent EIF2A phophorylation. **A) Schematic showing EIF2A kinases and their regulation.** Pathway agonists thapsigargin, poly (I∶C) and NaAS2O3 and their locus of action are indicated. **B) Effect of EIF2AK3/PERK ablation on EIF2A phosphorylation by CCT020312.** U-2OS human osteosarcoma cells were transfected with either of two different EIF2AK3/PERK siRNA oligonucleotides (PERK-1, PERK-2) or an irrelevant control (NT) for 72 hours. Cells were treated with 10 µM CCT020312 (CCT) or 2 µM thapsigargin (Tg) for the indicated times. Lysates were analysed by immunoblot as indicated. **C) Quantitation of PERK mRNA expression in siPERK transfected cells.** PERK mRNA was quantified in siRNA tranfected cells using SYBR Green based quantitative PCR. The comparative cycle threshold method was used to determine the fold change in PERK mRNA relative to cells tranfected with irrelevant oligonucleotide. GAPDH was quantified in parallel and used to normalise between samples. Bars represent the mean fold change in triplicate technical replicates. **D) Ablation of EIF2AK3/PERK prevents CCT020312-mediated cyclin D loss and accumulation of underphosphorylated pRB.** HCT116 human colon cancer cells were transfected with EIF2AK3/PERK siRNA oligo ‘2’ or non-targeting siRNA (NT). After 72 hours cells were treated with 10 µM CCT020312, 2 µM thapsigargin (Tg) or DMSO for times indicated. Cell lysates were analysed by immunoblotting as indicated.

EIF2A phosphorylation arises from activation of four different EIF2A kinases (EIF2AK1-4) that respond to distinct signalling cues (Figure 5A), [32], [33]. EIF2AK1/HRI is activated by heme accumulating in erythrocytes or oxidative stress, EIF2AK2/PKR by double-stranded RNA, EIF2AK3/PERK through an increase of unfolded proteins in the endoplasmic reticulum and EIF2AK4/GCN2 through deprivation of essential amino acids.

We tested for the involvement of these different EIF2AKs in the response to CCT020312. EIF2AK3/PERK depletion using small interfering RNA (siRNA) oligonucleotides strongly attenuated Ser51 EIF2A phosphorylation induced in response to CCT020312 or thapsigargin (Figure 5B, results quantified in Figure S6), and this was seen with two unrelated PERK targeting oligonucleotides. qPCR- based mRNA quantification (Figure 5C) revealed reduction of EIF2AK3/PERK mRNA in cells transfected with either oligonucleotide. Efficacy of depletion was higher in PERK-2 than PERK-1-transfected cells, consistent with the superior reduction of signalling seen in cells transfected with PERK-2 olignucleotide (Figure 5B). Knockdown of EIF2AK3/PERK also abolished cyclin D1 loss and the accumulation of underphosphorylated pRB arising subsequent to treatment with both agents, consistent with our hypothesis that these molecular events result from the activation of EIF2AK3/PERK signalling by CCT020312 or thapsigargin (Figure 5D, results quantified in Figure S6). Ablation of EIF2AK2/PKR by siRNA had no effect, although its knockdown inhibited EIF2A phosphorylation in response to poly (I∶C), a known activator of this EIF2AK (Figure S7). Likewise, addition of hemin, which inhibits EIF2AK1/HRI [34] and which suppressed EIF2A phosphorylation in cells exposed to oxidative stress, did not affect EIF2A phosphorylation in cells treated with CCT020312 (Figure S7). Together these observations clearly indicate the critical involvement of EIF2AK3/PERK in the phosphorylation of EIF2A, cyclin D loss and the attenuation of pRB phosphorylation seen in CCT020312-treated cancer cells.

### Selective stimulation of EIF2A signalling by CCT020312

**Figure 6:**
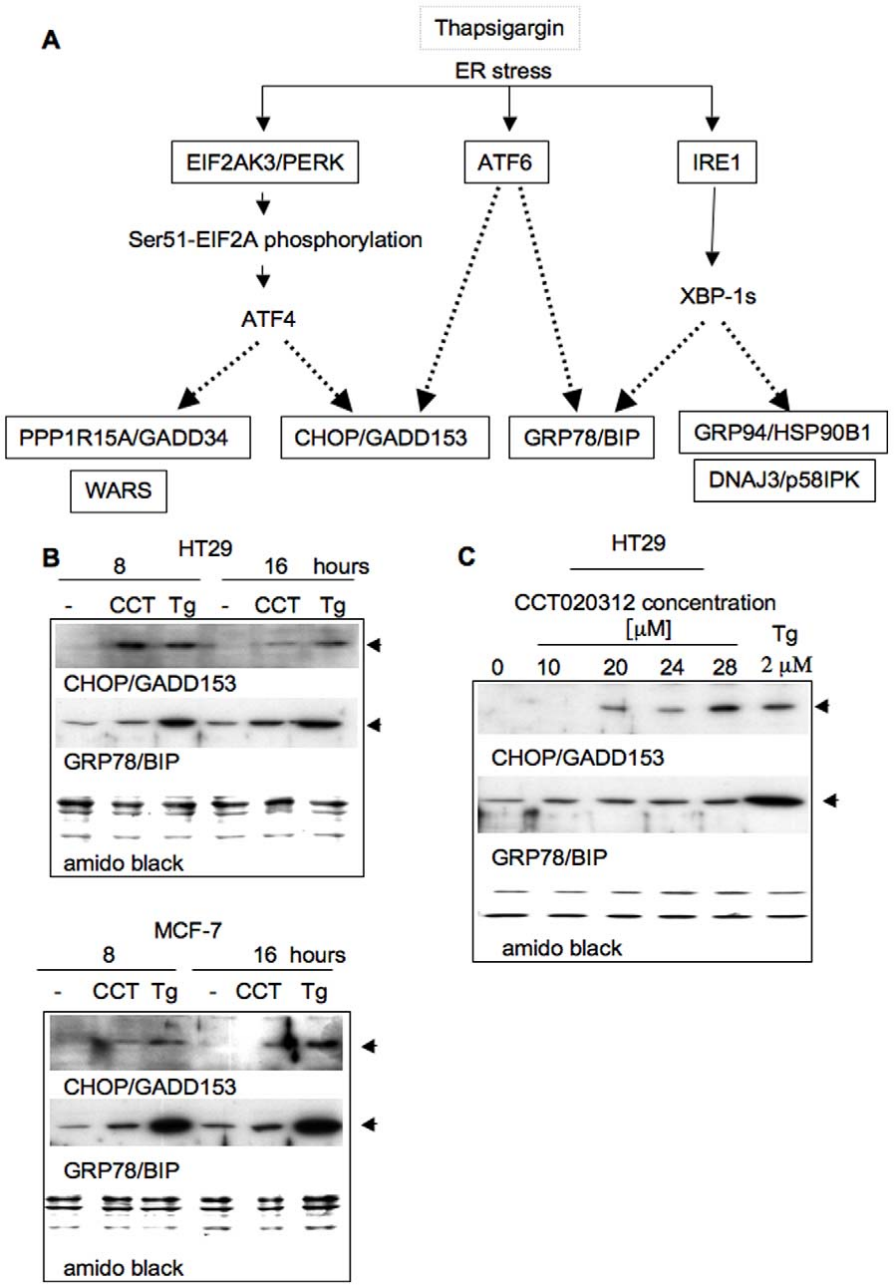
CCT020312 does not induce full ER stress signalling. **A) Schematic of unfolded protein response signalling.** Response biomarkers are indicated. **B) UPR response marker expression in HT29 colon and MCF7 breast cancer cells.** Cells were treated with CCT020312 (CCT, 10 µM) or thapsigargin (Tg, 2 µM) as indicated and analysed by immunoblotting for CHOP/GADD153 and GRP78/BIP. **C) UPR response marker expression following CCT020312 dose escalation.** HT29 cells were treated for 16 h. Lysates were analyzed as in B.

EIF2AK3/PERK activation and the consequent phosphorylation of EIF2A classically ensue as part of the unfolded protein response (UPR) [35]. The UPR is initiated when unfolded or misfolded proteins accumulate in the lumen of the endoplasmic reticulum, leading to EIF2AK3/PERK activation and, as a consequence, activating transcription factor 4 (ATF4) driven gene transcription, but also other signalling, unrelated to EIF2AK3/PERK (see Figure 6A). Thus, transcription factor ATF6 is released from its ER membrane position allowing translocation to the cell nucleus. Furthermore, protein kinase and nuclease inositol-requiring 1 (IRE1) is activated and facilitates mRNA processing to produce the spliced variant form of the X-box transcription factor XBP1 (XBP1s) [35].

To assess whether ATF6 and IRE1 are activated alongside EIF2AK3/PERK, CCT020312-treated HT29 colon cancer and MCF7 breast cancer cells were probed for markers downstream of these different signalling effectors. Consistent with its effect on Ser51-EIF2A phosphorylation, CCT020312-treatment of both cell types resulted in accumulation of the ATF4 target C/EBP homology protein/growth arrest and DNA damage 153 (CHOP/Gadd153) (Figure 6B). However, induction of the ATF6 target 78 kDa glucose-regulated protein/Binding immunoglobulin protein (GRP78/BIP) was virtually undetectable, even at late times (16 h, Figure 6B) or following treatment with higher concentrations of CCT020312 (Figure 6C). Induction of GRP78/BIP was readily detected in parallel cultures of these cells treated with thapsigargin. We also isolated mRNA from CCT020312 and thapsigargin-treated HCT116 and HT29 cells and probed for the presence of spliced XBP1 transcript using PCR (Figure S8). Spliced XBP1 mRNA was not detected in CCT020312-treated cells but was readily detected in thapsigargin-treated cells (Figure S8). As observed previously, EIF2A phosphorylation was readily increased following both treatments in parallel samples (Figure S8), indicative that CCT020312 as well as thapsigargin had both elicited EIF2AK activation. These results suggest that, in contrast to thapsigargin, CCT020312 does not act by triggering the full spectrum of UPR responses.

Inspection of mRNA expression patterns based on the cDNA microarray analysis described earlier supports this hypothesis (Figure S9). Expression of two genes, GRP94/HSP90B1 and DNAJ3/p58IPK, recently shown to be regulated during UPR through the IRE1 effector XBP1 [36] was not up-regulated in CCT020312-treated cells, although these genes were up-regulated in thapsigargin-treated cells (Figure S9). However, up-regulation of mRNA for CHOP/Gadd135 and the tryptophanyl-tRNA synthetase WARS, previously shown to depend on EIF2A phosphorylation [37], was seen with both treatments (Figure S9).

Confirmatory evidence for the absence of outright UPR also came from the analysis of primary mouse embryonic fibroblasts (MEFs), a cell model that has been used for establishing many of the concepts of UPR signalling transmission in mammals [35]. CCT020312 treatment of the MEFs led to CHOP induction, whilst accumulation of the small splice-variant of XBP1 (XBP1s) was not triggered. Rapid XBP1s accumulation was however seen when these cells were treated with thapsigargin (Figure S9). CCT020312 also suppressed cyclin D1 expression in these fibroblasts. These observations are consistent with the involvement of EIF2AK3/PERK activity in the CCT020312 elicited effects but strongly argue against induction of full UPR as the molecular mechanism by which CCT020312 acts.

**Figure 7:**
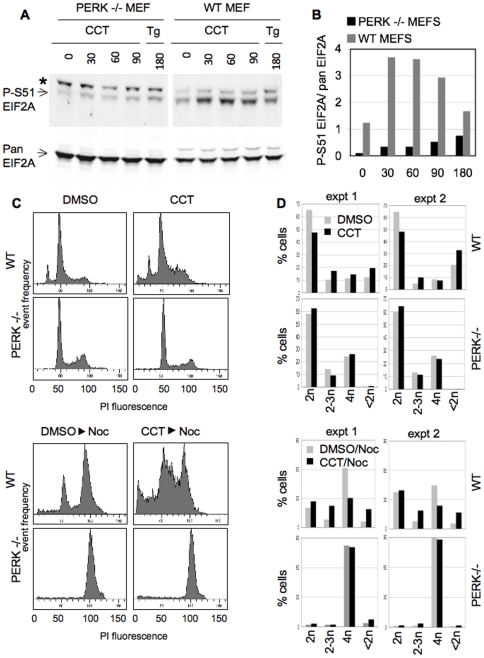
Effects of CCT020312 exposure on wild type and PERK KO MEFs. **A) EIF2A phosphorylation.** SV40-immortalised wild type (WT) and PERK KO MEFs (PERK−/−) were treated with 10 µM CCT020312 (CCT), 2 µM thapsigargin (Tg) for the time indicated. Cell lysates were analysed using P-S51 EIF2A and pan EIF2A selective antibody. The position of P-S51 EIF2A is indicated with an arrow beneath an unrelated, high molecular weight, non-specific band (*). B) **Signal quantification for data shown in A).** Charts depict the background corrected signal for P-S51 EIF2A relative to that of pan EIF2A in the same samples. Signals were quantified using Image J. **C) Cell cycle response.** MEFs were treated with 10 µM CCT020312 or vehicle (DMSO) for 30 hrs and analysed for cell cycle distribution. Where indicated, Nocodazole (Noc) was added to the culture medium at a concentration of 1 µg/ml for the final 16 hours. Raw propidium iodide profiles are shown. Cells were treated in parallel to those analysed for A. **D) Quantitative analysis of cell cycle distribution.** Experiments were as described for C). Charts depict the percentage of cells with DNA content as indicated, nocodazole was added where indicated. Results for two independently run experiments are shown.

To probe for the significance of PERK in the antiproliferative action of CCT020312, we used SV40 immortalised fibroblasts from wild-type (wt) and PERK knockout (PERK −/−) mouse embryos ([38]) (Figure 7). In keeping with our earlier results indicating EIF2AK3/PERK-dependence of the CCT020312-induced EIF2A phosphorylation we found EIF2A phosphorylation to be radically reduced in PERK −/− MEFs following exposure to either thapsigargin or CCT020312 (Figure 7A,B). Significantly, cell cycle analysis revealed a considerable distortion of the cell cycle profile in wt MEFs following CCT020312 treatment, with overt inhibition of progression into G2/M-phase, as revealed when a nocodazole-induced G2/M block was established following exposure of these wt MEFs to CCT020312 (Figure 7C, D). We note that these cells did not respond with G1-phase accumulation (2n) to CCT020312, consistent with the notion that this arrest is facilitated by pRB and its orthologues which in these MEFs are inactivated as a consequence of SV40 transformation. Instead they respond with increased S-phase accumulation (2−3n) and apoptosis, as revealed by the increase of cells with a sub G1 DNA content (<2n). Neither of these responses were evident in paired SV40-transformed PERK−/− MEFs (Figure 7C, D). Instead these PERK-modified cells were capable of moving into G2 with identical efficacy, irrespective of whether CCT020312 was present or absent, indicative that cell cycle transit in these cells is not affected by CCT020312. These results fully support the notion that selective activation of EIF2AK3/PERK signalling is the mode of action through which CCT020312 causes attenuation of cell cycle transit and inhibition of proliferation.

### Interaction of CCT020312 with paclitaxel

**Figure 8:**
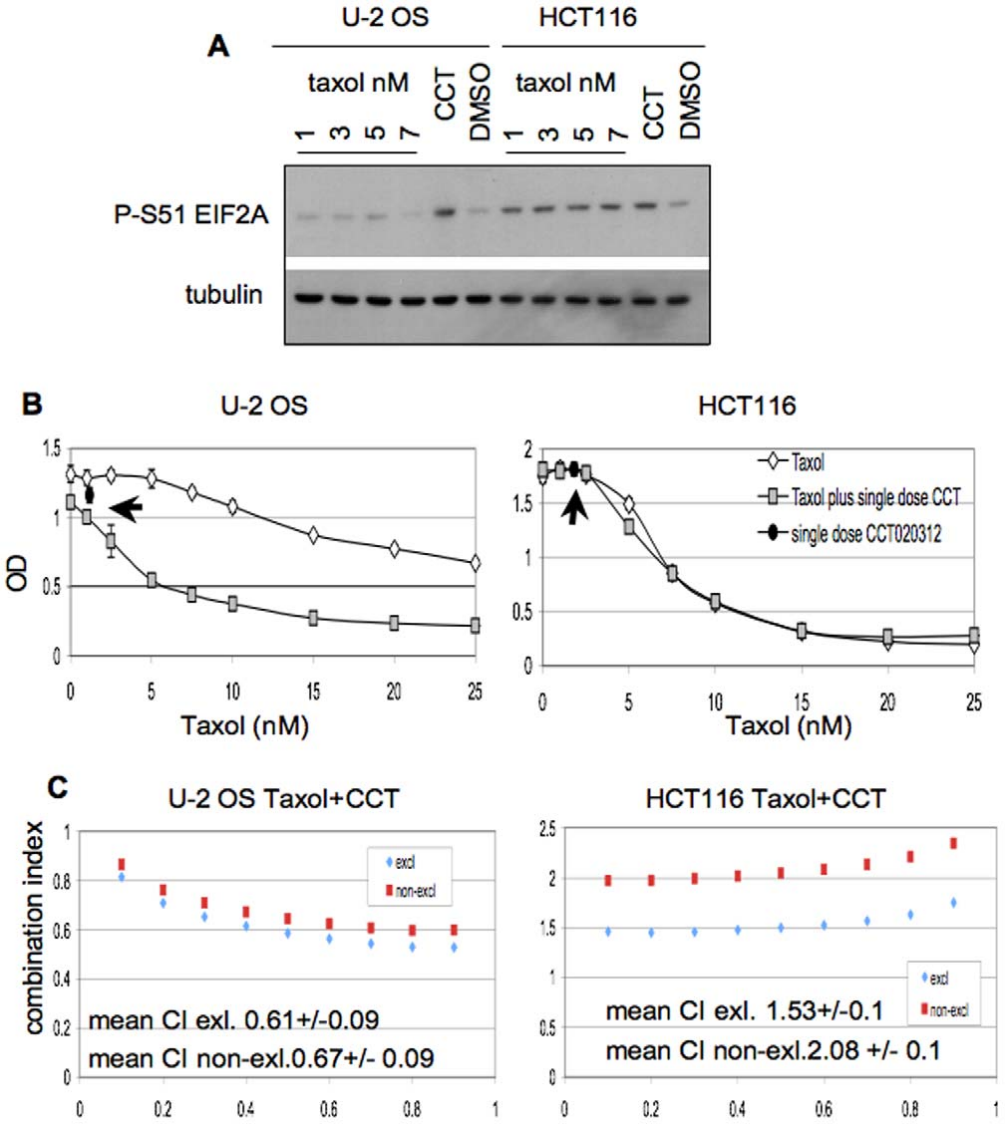
Interaction of CCT020312 with paclitaxel. **A) Paclitaxel-associated EIF2A phosphorylation in U-2OS and HCT116 cells.** U-2OS and HCT116 were exposed to 10 µM CCT020312 (CCT), DMSO or paclitaxel (taxol) for 4 hours. Cell lysates were analyzed by immunoblotting for P-S51-EIF2A and tubulin. **B) Proliferation inhibition by paclitaxel in the presence of CCT020312.** Cells were treated with increasing amounts (1–25 nM) of paclitaxel in the absence and presence of a fixed dose (2.5 µM) of CCT020312. Arrows denote proliferation inhibition in cells treated with 2.5 µM CCT020312 only. Cell lines were as indicated. **C) Multiple drugs effect analysis:** U-2OS and HCT116 cells were treated with escalating doses of paclitaxel, CCT020312 or their combination. Combination indices (CIs) for each dose are shown. The calculated mean CI and standard error is indicated. “CI excl” assumes agents act by a competing, mutually exclusive mechanism of action, “CI no-exl. assumes agents act through distinct, exclusive mechanisms of action [57].

Recent work indicates a link between ER stress-mediated EIF2AK3/PERK signalling and the cellular sensitivity to multiple anticancer agents, including taxanes [39], [40]. We therefore evaluated if CCT020312 could enhance the sensitivity of cells to paclitaxel. We treated U-2OS human osteosarcoma cells, which do not induce EIF2A phosphorylation in response to paclitaxel, and HCT116 colon cancer cells, in which EIF2A phosphorylation increases upon treatment with this drug (Figure 8A), with increasing concentrations of paclitaxel, either alone or in combination with CCT020312. Exposure of both these cell types to 2.5 µM CCT020312 resulted in a clear augmentation of the paclitaxel-induced growth inhibition in U-2OS cells but had no effect in HCT116 cells (Figure 8B). Fixed ratios of paclitaxel and CCT020312 across a 25-fold range yielded combination indices that were consistently less than one (<1) in U-2OS, indicating a greater than additive interaction of these two agents using median effect analysis [41]. In contrast, combination indices of greater than one (>1) were obtained in HCT116 cells, indicating a less than additive interaction between CCT020312 and paclitaxel in these cells (Figure 8C and Figure S10). Experiments in cells with taxane resistance due to increased multidrug resistance (MDR1) P-glycoprotein expression did not reveal synergy between CCT020312 and paclitaxel, nor did the ablation of p53, thought to mediate apoptosis in HCT116 cells, cause cooperation (not shown). This suggests that a selective complementation of EIF2AK3/PERK signalling deficiency underlies the cooperation between CCT020312 and paclitaxel seen in U2-OS osteosarcoma cells. Thus in addition to its ability to confer G1/S checkpoint activation and proliferation control, CCT020312, or agents like it, may hold promise in conferring sensitization of cancer cells in specific situations of paclitaxel resistance.

## Discussion

We previously described a mechanism-based screening approach to isolate G1/S checkpoint activating agents [20]. Using this approach we have identified several structurally unrelated chemical series, including CCT020312, and report here the mechanism of action by which this agent achieves checkpoint activation in cells. Our analysis indicates that CCT020312 promotes EIF2A phosphorylation with the known consequence of D-type cyclin depletion and a loss of ability to phosphorylate pRB. Our results identify loss of cyclin D expression as a major and early event in CCT020312-treated cells and show this, along with inhibition of cell cycle transit by CCT020312 is a consequence of EIF2AK3/PERK signalling. Together these results identify EIF2AK3/PERK signalling as the target mechanism through which CCT020312 achieves G1/S checkpoint activation, corroborating earlier evidence that link this signalling pathway to the control of CDK4/6 activation in mammalian cells [30].

EIF2AK3/PERK activation forms part of the UPR that arrests cell proliferation following accumulation of unfolded proteins in the ER. However UPR signalling involves other events in addition to EIF2AK3/PERK activation, including membrane release and activation of ATF6 and IRE1 [42] leading to activation of a complex transcriptional response both independent of, and collaborative with, EIF2AK3/PERK activation. Our analysis indicates that CCT020312 does not induce responƒses that ensue from activation of IRE1 or ATF6, although it effectively stimulates EIF2A phosphorylation and associated downstream signalling linked to this event. Hence, our results show that CCT020312 does not elicit UPR, but that it acts selectively to instigate proliferation inhibition through an EIF2A phosphorylation-associated translation inhibition. While EIF2AK3/PERK activation is consistently seen with agents that disable ER function, these agents classically trigger full UPR. To our knowledge agents with selectivity for eliciting EIF2AK3/PERK signalling alone have not been described previously.

While the precise way through which CCT020312 elicits EIF2AK3/PERK signalling has not yet been elucidated, the current knowledge as to how activation of this kinase is achieved implies a narrow set of options. EIF2AK3/PERK auto-activates through oligomerization-induced autophosphorylation [43], [44]. Initiation of EIF2AK3/PERK oligmerization is thought to follow from competition between unfolded ER protein clients and EIF2AK3/PERK for the chaperone heat shock 70 kDa protein 5 (HSPA5)/GRP78/BIP, which associates with and prevents oligomerization of the kinase. However, HSPA5/GRP78/BIP also controls ATF6 and IRE1 [45], which according to our analysis are not activated by CCT020312. Hence outright interference with HSPA5/GRP78/BIP functioning cannot explain the mechanism of action of CCT020312. More conceivable is a mode of action whereby CCT020312 interacts with EIF2AK3/PERK, either preventing the association of this kinase with HSPA5/GRP78/BIP or promoting HSPA5/GRP78/BIP-resistant oligomerization. It is also possible that CCT020312 prevents feedback regulation that silences EIF2AK3/PERK signalling following activation by spurious ER stress as part of normal cell homeostasis. Feedback regulation during UPR involves enhanced production of protein phosphatase 1 regulatory subunit 15A (PPP1R15A)/GADD34 consequent to ATF4 activation (see Figure 6A), which in turn recruits the serine/threonine-protein phosphatase PP1 to dephosphorylate EIF2A [46]. A previous chemical biology approach identified salubrinol as an inhibitor of PP1-mediated EIF2A dephosphorylation. Like CCT020312, salubrinol provokes upregulation of CHOP/GADD153, but does not initiate XBP1 splicing or GRP78/BIP production [47]. However, and in contrast to CCT020312, salubrinol induces Ser51 phosphorylation of EIF2A in EIF2AK3/PERK-negative backgrounds, indicating that the mechanism of action of salubrinol and that of CCT020312 are mechanistically distinct.

We demonstrate potent antiproliferative activity of CCT020312 at low µmolar concentrations, which is detectable in both p53 positive (HCT116) and p53 negative (HT29) human colon cancer cells and remains considerable even under conditions of time-limited exposure to the compound. The known consequences of EIF2AK3/PERK activation are consistent with these observations. While the initial result of EIF2AK3 activation is acute inhibition of protein translation, leading to G1 restriction point activation and arrest, prolonged activation is linked to loss of cell viability, conceivably due to an inability to produce anti-apoptotic proteins, which many cancer cells rely upon [48], [49]. The well-recognized dependence of cancer cells on anti-apoptotic functions may constitute a cancer-selective therapeutic window for the treatment with agents such as CCT020312. Recent work has implied the involvement of ER stress signalling in mediating the potency of several unrelated cytotoxic agents including, paclitaxel fluorouracil and cisplatin [39], as well as a supporting role of EIF2A phosphorylation in the response to anticancer agents targeting the ubiquitin/proteasome pathway [50], [51], indicating added utility for agents such as CCT020312 that are able to activate this form of signalling. In agreement with this hypothesis, we have demonstrated the ability of CCT020312 to sensitize cells that lack paclitaxel-mediated EIF2A phosphorylation response to the cytotoxic effects of paclitaxel. Apart from its intrinsic antiproliferative mode of action, CCT020312 and agents like it may be suitable for use in combination with paclitaxel and potentially other anticancer agents. Furthermore, EIF2AK3/PERK signalling has been found to alleviate beta-amyloid associated neurotoxicity [52] and reduce brainstem motor neurone death in a murine model of sleep apnea [53], suggesting that EIF2AK3/PERK pathway activation could be a potential target for therapeutic fields other than cancer, including conditions of hypoxia-associated neurotoxicity and neurodegenerative disorders such as Alzheimers disease. CCT020312 could serve as a mechanistic tool for proof-of-concept explorative experimentation in these various contexts and may provide a candidate for chemical optimization.

## Materials and Methods

### Cell culture and agent treatment

Cell lines were obtained from ATCC and maintained in DMEM or RPMI (GibcoBRl) containing 10% FCS. Primary MEFs were isolated from day 13.5 embryos. T antigen immortalised PERK +/+ and −/− MEFs were kindly provided by the Ron laboratory. MEFs were maintained in DMEM Supplemented with 10% FCS, 5 mM glutamine, antibiotics, 1× MEM non-essential amino acids and 55 mM 2-mercaptoethanol. Unless stated otherwise cells were seeded at subconfluent density and cultured for 24 h prior to agent treatment. CCT020312 was dissolved in DMSO at a concentration of 10 mM and stored at −70°C. Thapsigargin (Sigma) was dissolved in DMSO at a concentration of 2 mM and used at the indicated concentrations to induce EIF2AK2/PERK-mediated EIF2A phosphorylation. Poly (I∶C) and sodium arsenite (NaAS2O3, Sigma, aqueous solution) were employed at the indicated concentrations to induce EIF2A phosphorylation by EIF2AK2/PKR and EIF2AK1/HRI, respectively. Hemin chloride (Sigma) was dissolved in DMSO and used at the indicated concentrations to block EIF2AK1/HRI activity in cells. All agents were made up in growth medium, 10% FCS at their respective working concentrations immediately prior to addition to the adherent cells. To monitor cyclin D1 protein stability cells were treated with 20 µg/ml cycloheximide in the absence or presence of CCT020312.

### Compounds and Compound screening

The Cell-based immunoassay for the detection of pRB-P-Ser608 has been described [20]. The assay used a mouse monoclonal antibody that recognizes the phosphorylated form of Ser 608 on pRb on a fixed monolayer of cells seeded in a 96 well format in combination with a Europium-labelled secondary antibody for signal detection. Signal detection was by a time-resolved fluorescence. The Cancer Therapeutics Unit internal compound collection (>63.000 compounds), comprising a selection of diverse small molecules either purchased from commercial suppliers, archived in-house or obtained through Cancer Research UK was screened in a 96 well format using conditions essentially as described in [20]. Compounds were used at 10 µM with assay endpoint at 24 h following compound addition. To identify candidate hits, data were filtered for reduction of signal by 50% or greater compared to the means of quadruple DMSO controls run in parallel in the same plate. Values were normalized to protein retained in the wells at the assay endpoint. All plates passed the quality control criteria of Z factor >0.4 [54]. Hits were confirmed using the screening assay and positives further validated by immunoblot using pan pRB antibody (14001A, Pharmingen) and antibody selective for Ser608 unphosphoryated pRB (14441A, Pharmingen). Preparation of CCT020312 and generation of analogues is described below. Structural integrity and purity of compounds including the original hit matter was established and confirmed using mass spectrometry.

### Cell lysate preparation, immunoblot analysis and antibodies

Cell lysates for immunoblot analysis were prepared in 50 mM Hepes-KOH pH 7.4, 250 mM NaCl, 5 mM EDTA, 0.5% Triton, 10 mM-glycerophosphate, 10 mM NaF, 1 mM NaVO3, 1 mM DTT, 1 mM PMSF, 1% aprotinin, 2.5 µg/ml leupeptin). Lysates were cleared by centrifugation at 10,000× g for 10 minutes at 4°C. Protein content was estimated by Bradford assay (Bio-Rad). Immunoblots were performed using standard procedures. Protein antibody complex was detected using horseradish peroxidase conjugated secondary antibody, followed by enhanced chemiluminescence ECL according to the manufacturer's instructions (GE healthcare). Primary antibodies used were; anti pRB-phospho-Ser608 antibody (P-S608-pRB, Barrie et al. 2003) pan pRB (14001A, Pharmingen), non-phospho-Ser608 pRB (NP-pRB, 14441A, Pharmingen), Cyclin D1 (554181, Pharmingen), anti pRB Phopsho-Ser780 (P-S780-pRB, a gift from Onyx Pharmaceuticals), anti-pRB-phospho-Ser807/811 (P-S807/811-pRB, Sigma), Cyclin D3 (C-16, Santa Cruz), Cyclin E (14591c, Pharmingen), Cyclin A (BF683, Santa Cruz), CDK4 (H-22, Santa Cruz), CDK6 (C-21 Santa Cruz), CDK2 (M2, Santa Cruz), p27^KIP1^ (C19, Santa Cruz), phospho-Ser473 AKT (Cell Signalling), AKT (Cell Signalling), anti EIF2A (Cell Signaling), phospho-Ser51 EIF2A (P-S51-EIF2A, Cell Signalling), HSP70 (W27, Neomarkers), GADD153/CHOP (F-168, Santa Cruz), GRP78/BIP (H-129, Santa Cruz), EIF2AK2/PKR (Cell Signalling) and β-tubulin (Neomarkers).

### siRNA-mediated gene silencing

Cells were transfected with siRNA oligonucleotide at 20 nM using the HiPerFect lipid reagent (Qiagen) as per the manufacturer's published guidelines for the transfection of adherent cell lines. Oligonucleotides used for EIF2AK3/PERK knockdown were 5′-uaa acc guu aua cag uuu gtg-3′ (EIF2AK3-1) and 5′-uua acu ucu cgc auu acc utt-3′ (EIF2AK3-2). For EIF2AK2/PKR the Dharmacon Smartpool (M-003527-00) was used.

### Generation of recombinant cyclin/CDK enzymes and phosphorylation activity assay

Kinases were produced in Spodoptera frugiperda (SF9) insect cells. Cells were infected with pairs of cyclin and CDK-encoding baculoviruses, and kinases produced as recently described [55]. Purified baculovirus-produced Cyclin B/CDK1 was purchased from Upstate Biotechnology. Purified baculorvirus-produced Cyclin D1/CDK4 was a gift from Onyx Pharmaceuticals. Test tube reactions were assembled containing 50 mM HEPES-KOH, pH 7.4, 10 mM MgCl_2_, 10 mM MnCl_2_, 1 mM EDTA, 10 mM B-glycerophosphate, 0.1 µM protein kinase A inhibitor, 1 mM DTT, 1 mM PMSF, 1% aprotinin, 2.5 µg/ml leupeptin, with 10 µM ATP and 0.5 µCi [gamma −32P] ATP and 10 µg/ml Gluthathione-S-transferase (GST) fused pRB fragment, aa 792–928 (GST-pRB-ct) as the substrate. Compounds were added to the reaction prior to enzyme addition. Final reaction volumes were 50 µl and the reaction time 15 min at 30°C. Reactions were stopped using SDS containing sample buffer and analyzed using denaturing polyacrylamide gel electrophoresis with subsequent autoradiography. For plate-based kinase activity assays Immulon^R^ plates (Thermo Scientific) were coated with 1 µg per well of GST-pRB-ct in PBS. Plates were washed 3 times in PBS, 0.1% Tween 20 prior to use. Reactions were assembled containing 50 mM HEPES pH 7.4, 10 mM NaF, 1 mM sodium orthovanadate, 1 mM DTT, 10% glycerol, 25 µM ATP, 10 mM MgCl_2_ and enzyme. Compounds were added prior to addition of enzyme. Reactions were carried out for a set time at 30°C and stopped by adding 50 µl cold 0.2 M EDTA. Substrate phosphorylation was quantified using phosphorylation-selective anti-pRB antibody detection involving europium labelled secondary antibody (Perkin Elmer). Phopsho-Ser780 pRB was used for quantification of CDK4 activity, phospho-Ser807/811 pRB for the quantification of CDK1 activity.

### Cell cycle analysis and proliferation assays

Cell cycle analysis was performed using standard procedures and as described [56]. Propidium Iodide (PI) staining was used to determine the DNA content and Bromodeoxyuridine (BrdU) pulse labelling to assess DNA synthesis activity. The proliferation activity of cells was quantified using a standard sulphorhodamine B (SRB) colorimetric assay in a 96-well assay format. GI50 values were calculated by non-linear regression using the Prism V4.0 software. Multiple drug effect analysis was performed as described [41]. Combination index (CI) values were derived from parameters of the median effects plots. Statistical tests (unpaired, two-tail Student t test) were used to determine whether the mean CI values at multiple concentrations were significantly different from CI = 1.

### RNA preparation and transcript analysis

RNA for microarray analysis was generated by growing 0.7×10^6^ HT29 cells in 20 cm tissue culture dishes. After 48 h growth cells were exposed to either 7 µM CCT020312, 220 µg/ml Poly (I∶C) or 2 µM thapsigargin for various times. Floating and adherent cells for each time point were pooled and processed. Total RNA was isolated using RNACell (Applied Biossytems) on the ABI 6100 nucleic acid prepstation, following the manufacturer's instructions. The CyScribe Post-Labeling Kit (GE Healthcare) was used to label 5 µg of total RNA according to the manufacturer's recommendations. Reference samples were labelled with cy5 and the drug-treated test samples with cy3. Labelled test and reference samples were mixed and hybridised to the CRUK Human Whole Genome-wide Array v1.0.0 printed on type 7* slides (GE Healthcare). Arrays were hybridised for 3 days at 42°C.

The cy3 and cy5 signals were detected and visualized using a GenePix 4000B scanner and software (Axon Instruments, Foster City, CA, USA). Spots were curated and filtered for quality by automated spot flagging. Spots were assigned a good flag if the signal intensity was 1 standard deviation above background in 75% of the pixels in either channel, with a signal saturation of less then 3% in either channel. The output of the automated flagging was inspected visually and adjusted when necessary. The GenePix raw data files were analysed in Genespring (Agilent Technologies) using the “per–spot” and “per-chip” intensity–dependent Lowess normalization. A Lowess curve was fit through the log-intensity versus log-ratio plot. 20% of the data was used to calculate the Lowess fit at each point. This curve was used to adjust the control value for each measurement. Only genes that fulfilled the good flag criteria in at least 67% of the samples were used for analysis. Replicate samples were averaged. Genes were considered changed when a 1.5 fold change was observed relative to untreated controls for 2 or more time points. Genes that were modified by DMSO treatment were removed from the analysis. Hierarchical clustering was performed using Euclidian distance as the metric.

RNA for quantitative PCR analysis was prepared from cells in 6-well plates using RNeasy (Qiagen) assuming 1×10^6^ cells/well and using the manufacturer's ‘Animal Cells Spin’ protocol and QIAshredder option. Quantitative PCR was performed by a 2-step protocol involving a round of first strand cDNA synthesis and PCR amplification in the presence of SYBR green (Qiagen). Ready-To-Go T-Primed First-Strand reactions (GE Healthcare) were used for first-stand synthesis. The Quantitect SYBR Green PCR kit and Quantitect validated primer pairs for EIF2AK3/PERK and GAPDH and Cyclin D1 (Qiagen) were employed to quantify these transcripts in first-strand samples using an ABI Prism 7700.

Northern blots were performed using standard formaldhyde agarose gel electrophoresis. Probes for hybridization were prepared by PCR and labelled using the BioPrime DNA Labelling System (Invitrogen). Hybridizatrion and detection followed the manufactorer's instructions.

### CCT020312 synthesis and analogues

The synthetic route for CCT020312 and analogues is provided in Supplemental Methods and Materials S1.

## Supporting Information

Figure S1 **Effect of CCT020312 on cyclin/CDK activity in vitro. A), B) ELISA-based activity assay.** Enzyme activity was determined using 96 well microtitre plates coated with 1 µg per well of GST-pRB-ct. Reactions contained CCT020312 or the pan CDK inhibitor flavopiridol [6], as indicated. CDK4 activity was detected using rabbit anti-pRB-P-Ser780 (1∶3000), CDK1 activity was detected using rabbit anti-pRB-P-Ser807/811 (Sigma 1∶5000) followed by europium-labelled anti-rabbit 2° antibody (Perkin Elmer, Life Sciences, 0.1 mg/ml). Signals were detected by time-resolved fluorescence. Curve fit for representative assay (A). Calculated IC50 average, n = 3 (B). **C)** *In vitro* phosphorylation assays. Baculovirus-infected SF9 lysates directing the expression of CyclinD1/CDK4, Cyclin E/CDK2 or Cyclin A/CDK2 complexes were added to a kinase reaction containing 0.5 mg GST-pRB-ct as substrate and 10 µM ATP. CCT020312 was added at the concentrations indicated. K4 and K2 indicate reactions run in the presence of catalytic subunits CDK4 or CDK2 only. The CDK1/2 selective inhibitor R-roscovitine was included as a positive control. Reactions were analysed by SDS gel electrophoresis followed by autoradiography. **Reference.** 6. Senderowicz AM (1999) Flavopiridol: the first cyclin-dependent kinase inhibitor in human clinical trials. Invest New Drugs 17: 313–320.
(TIF)

Figure S2 **Concentration-response relationship for inhibition of pRB phosphorylation and proliferation. A) Concentration-response curve for reduction of pRB phosphorylation in HT29 and HCT116 colorectal carcinoma cells.** pRB phosphorylation was quantified using immunoadsorbance as employed for the primary screen. Signals normalized to protein content in respective wells are shown. Error bars represent the standard deviation based on three replicate experiments. **B) Concentration-response curve for growth inhibition in HT29 and HCT116 colorectal carcinoma cells.** Proliferation was quantified 96 hours post compound addition using a sulphorhodamine B based colorimetric assay. **C) Growth inhibition following pulse-treatment of cells.** Cells were treated for the time indicated after which compound containing medium was removed. Proliferation was quantified 96 hours post treatment using a sulphorhodamine B based colorimetric assay. GI50 calculations were performed by non-linear regression using the Prism V4.0 software.
(TIF)

Figure S3 **Effects of CCT020312 on expression of G1/S CDKs and their regulators. A) Changes in biomarker expression over 24 h**. HT29 cells were treated with 10 µM CCT020312 for the periods indicated. Lysates were prepared at each time point and analysed as in FIG. 2E. **B) Loss of cyclin D1 is widely detected in cell lines treated with CCT020312**. Cell lines as indicated were treated with 10 µM CCT020312 (+) or vehicle (−) for 6 h. Lysates were analyzed for cyclin D1 expression by immunoblot. Membrane staining with amido black (A, D) or probing for tubulin (B, C) was used to reveal loading.
(TIF)

Figure S4 **Effect of CCT020312 on cyclin D1 turnover and mRNA accumulation. A) CCT020312 does not affect Cyclin D1 stability**. MCF-7 were treated with 20 µg/ml cycloheximide in combination with 10 µM CCT020312, as indicated. Lysates were analysed by Immunoblotting using anti Cyclin D1 and tubulin. A′) quantification of Cyclin D decay using cy-5 conjugated secondary antibody. Signals were quantified by PhosphoImager. **B) CCT020312 does not trigger Thr286 phosphorylation of Cyclin D1**. MCF-7 cells were treated with 10 µM CCT020312 (+) for the times indicated. Lysates were adjusted for protein content and analysed undiluted or diluted 1∶2 by immunoblotting blotting using antibodies as indicated. MCF-7 cells were treated with 100 µM etoposide a known inducer of cyclin D-Thr286 phosphorylation (B). **C), D) Effect of CCT020312 on cyclin D1 steady mRNA state levels.** MCF-7 cells were treated with 10 µM CCT020312 for the time indicated. RNA was analysed by SYBR green assisted q PCR (C), or Northern blot (D). Error bars in C represent the means of four parallel technical replicates. qPCR reactions were evaluated using the standard curve method. GAPDH was used as a reference for normalization.
(TIF)

Figure S5 **EIF2A phosphorylation following CCT020312 treatment.** Signal quantification for results shown in Figure 4A. Electronic scans were produced from primary autoradiograms and analysed using ImageJ (http://rsbweb.nih.gov/ij/). Charts depict background corrected signal quantities for P-S51 EIF2A relative to the signal quantities for pan EIF2A in the same samples.
(TIF)

Figure S6 **Effect of EIF2AK3/PERK ablation on CCT020312-mediated EIF2A phosphorylation and loss of cyclin D and pRB phosphorylation.** A) **Signal quantification for results shown in** **Figure 5B** **.** Charts depict background corrected signal for P-S51 EIF2A relative to that of pan EIF2A in the same samples. B) **Signal quantification for results shown in** **Figure 5C** **.** Charts depict background corrected raw signal quantities for Cyc D1, nonphosphorylated RB and pan RB. Quantification was performed using electronic scans produced from primary autoradiograms. Data were analysed using ImageJ (http://rsbweb.nih.gov/ij/).
(TIF)

Figure S7 **Impact of EIF2AK inhibition on CCT020312 signalling. A) EIF2AK2/PKR ablation fails to affect CCT020312 mediated EIF2A phosphorylation.** U2-OS human osteosarcoma cells were transfected with 20nM siRNA targeting PKR, or a control. Following 72 hours of RNAi cells were treated with 10 µM CCT020312 or DMSO for the indicated times, or with 500 µg/ml Poly (I∶C). Cells were harvested and lysates analysed by immunoblotting using antibodies as indicated. **B) EIF2AK1/HRI inhibition fails to inhibit CCT020312-mediated EIF2A phosphorylation.** U2-OS cells were treated with 0, 40 or 80 µm of the EIF2AK1/HRI inhibitor hemin for 1 h. Hemin-containing media was replaced with media containing 10 µM CCT020312 or 200 µM sodium arsenide (AS) or DMSO. Cells were lysed after 30 min and lysates analysed by immunoblotting as indicated. **(B) Regulation of genes responsive to integral stress response signalling.** mRNA abundance for CHOP/GADD153 and WARS, previously shown to depend on stress signalling involving EIF2A phoshorylation, as determined from cDNA data on thapsigargin and CCT020312 treated cells. Expression values shown are relative to DMSO treated control samples. mRNA expression data were extracted from the microarray dataset. Normalized data are expressed as a ratio of treated cells over untreated control cells. **C) Activation of EIF2A but not UPR selective signalling in mouse embryo fibroblasts (MEFs).** Primary MEFs were treated with 10 µM CCT020312 or 2 µM thapsigargin for 8 hours and analysed by immunoblot using antibodies to detect markers of EIF2AK (ATF4 and CHOP) and IRE involving (XBP1s) signalling.
(TIF)

Figure S8 **Absence of detectable XBP1 splicing in CCT020312 treated human cancer cells. A) PCR based detection of XBP1 splicing.** HT29 or HCT116 cells were treated with 10 µM CCT020312 (CCT) or 2 µM thapsigargin (Tg) for the time indicated. Alternative splicing of XBP1 was detected in total RNA extracts from these cells by reverse transcription PCR. **B) Detection of EIF2A phosphorylation.** Extracts for protein analysis were generated in parallel to RNA preparations analysed for A. **C) Signal quantification for results shown in B).** Charts depict background corrected P-S51 EIF2A after normalization against pan EIF2A. Signals were quantified as for supplemental Figure 5 using Image J.
(TIF)

Figure S9 **CCT020312 fails to trigger unfolded protein response selective signalling. A) Regulation of chaperone encoding genes activated downstream of XBP1.** mRNA abundance for GRP94/HSP90B1 and DNAJ3/p58PK, previously shown to rely on XBP1 signalling [7] as determined from cDNA microarray data on thapsigargin and CCT020312 treated cells. Expression values shown are relative to DMSO treated control samples. mRNA expression data were extracted from the microarray dataset. Normalized data are expressed as a ratio of treated cells over untreated control cells. **(B) Regulation of genes responsive to integral stress response signalling.** mRNA abundance for CHOP/GADD153 and WARS, previously shown to depend on stress signalling involving EIF2A phoshorylation [8], as determined from cDNA data on thapsigargin and CCT020312 treated cells. Expression values shown are relative to DMSO treated control samples. mRNA expression data were extracted from the microarray dataset. Normalized data are expressed as a ratio of treated cells over untreated control cells. **C) Activation of EIF2A but not UPR selective signalling in mouse embryo fibroblasts (MEFs).** Primary MEFs were treated with 10 µM CCT020312 or 2 µM thapsigargin for 8 hours and analysed by immunoblot using antibodies to detect markers of EIF2AK (ATF4 and CHOP) and IRE involving (XBP1s) signalling. **References.** 7. Lee AH, Iwakoshi NN, Glimcher LH (2003) XBP-1 regulates a subset of endoplasmic reticulum resident chaperone genes in the unfolded protein response. Mol Cell Biol 23: 7448–7459. 8. Mori K (2009) Signalling pathways in the unfolded protein response: development from yeast to mammals. J Biochem 146: 743–750.
(TIF)

Figure S10 **Interaction of CCT020312 and paclitaxel.** A, B) Cell proliferation activity of cells following treatment with paclitaxel, CCT020312 or the combination thereof, as determined using a 96 hour sulphorhodamine B (SRB) colorimetric assay run in a 96-well assay format. Agent concentrations were as indicated. Data were used to calculate combination indices for Figure 8C.
(TIF)

Table S1 **Prediction of small molecule agents connected to the mechanism of action of CCT020312.** The top ten hits are shown. Analysis output based on cmap 02 (http://www.broadinstitute.org/cmap/http://www.broadinstitute.org/cmap/).
(DOC)

Results S1 Assessment of hit matter.
(DOC)

Methods and Materials S1 XBP1 splicing and General Methods for the preparation of compounds.
(DOC)
