# Supplementary figures and images for: Mechanism-Based Screen for G1/S Checkpoint Activators Identifies a Selective Activator of EIF2AK3/PERK Signalling
Source: PLoS One. 2012 Jan 12;7(1):e28568. doi: 10.1371/journal.pone.0028568 (PMC3257223; doi:10.1371/journal.pone.0028568)

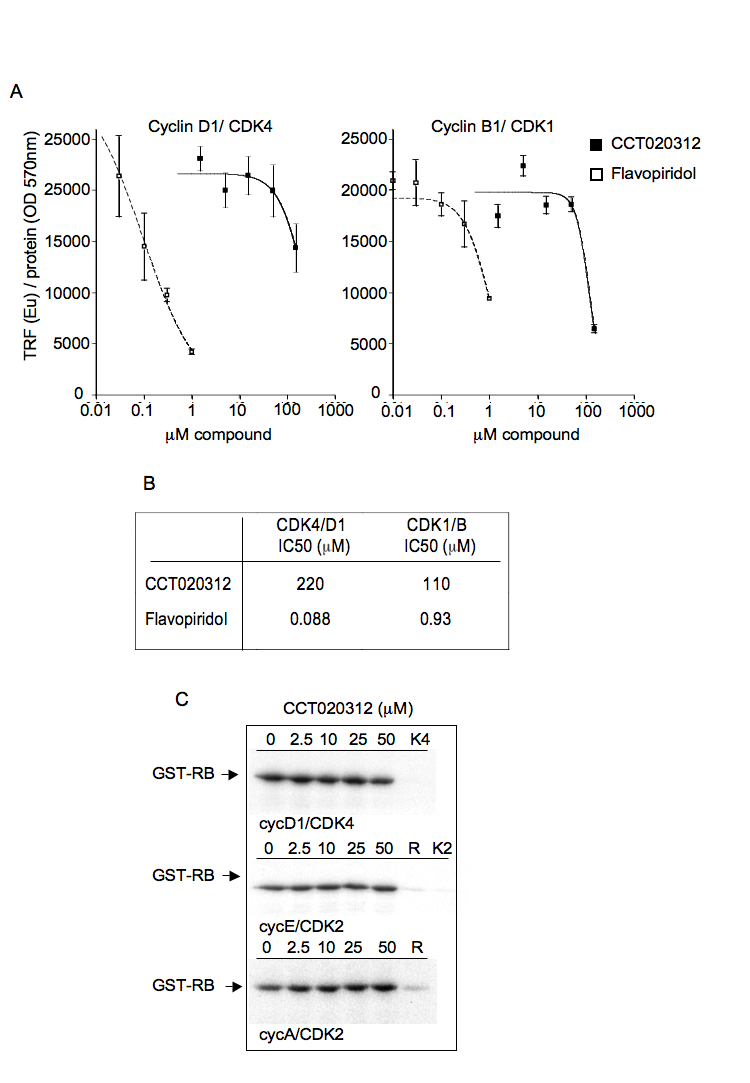

Supplement: Figure S1 — Effect of CCT020312 on cyclin/CDK activity in vitro. A), B) ELISA-based activity assay. Enzyme activity was determined using 96 well microtitre plates coated with 1 µg per well of GST-pRB-ct. Reactions contained CCT020312 or the pan CDK inhibitor flavopiridol [6], as indicated. CDK4 activity was detected using rabbit anti-pRB-P-Ser780 (1∶3000), CDK1 activity was detected using rabbit anti-pRB-P-Ser807/811 (Sigma 1∶5000) followed by europium-labelled anti-rabbit 2° antibody (Perkin Elmer, Life Sciences, 0.1 mg/ml). Signals were detected by time-resolved fluorescence. Curve fit for representative assay (A). Calculated IC50 average, n = 3 (B). C) In vitro phosphorylation assays. Baculovirus-infected SF9 lysates directing the expression of CyclinD1/CDK4, Cyclin E/CDK2 or Cyclin A/CDK2 complexes were added to a kinase reaction containing 0.5 mg GST-pRB-ct as substrate and 10 µM ATP. CCT020312 was added at the concentrations indicated. K4 and K2 indicate reactions run in the presence of catalytic subunits CDK4 or CDK2 only. The CDK1/2 selective inhibitor R-roscovitine was included as a positive control. Reactions were analysed by SDS gel electrophoresis followed by autoradiography. Reference. 6. Senderowicz AM (1999) Flavopiridol: the first cyclin-dependent kinase inhibitor in human clinical trials. Invest New Drugs 17: 313–320. (TIF) [file pone.0028568.s001.tif]

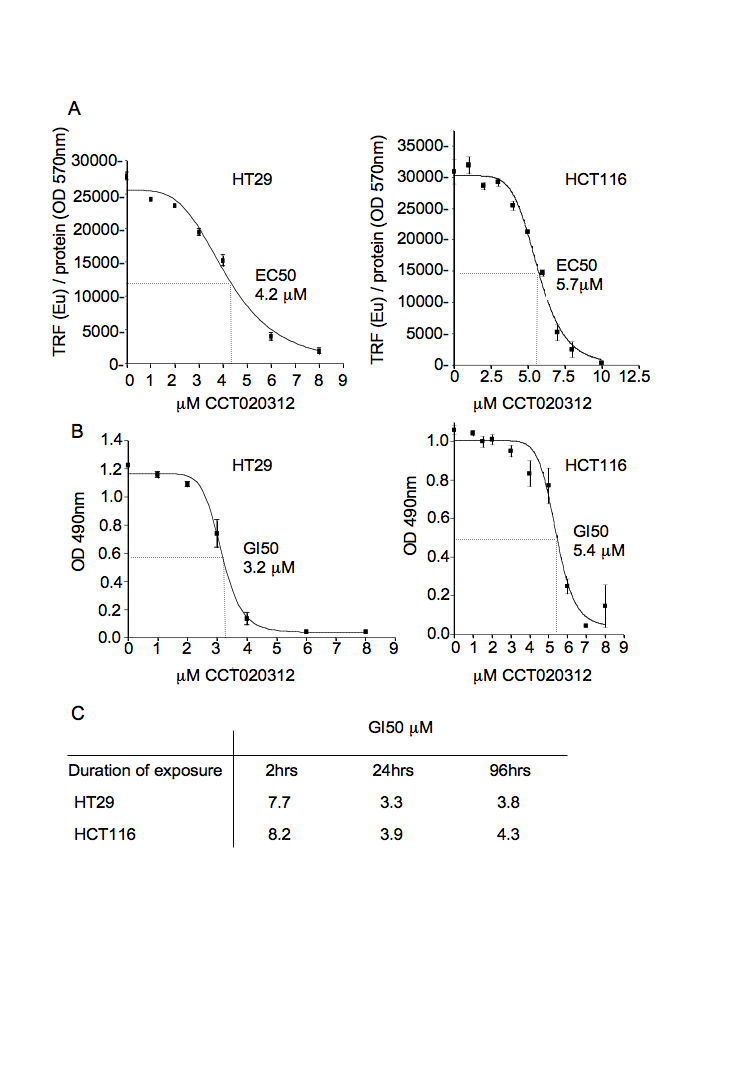

Supplement: Figure S2 — Concentration-response relationship for inhibition of pRB phosphorylation and proliferation. A) Concentration-response curve for reduction of pRB phosphorylation in HT29 and HCT116 colorectal carcinoma cells. pRB phosphorylation was quantified using immunoadsorbance as employed for the primary screen. Signals normalized to protein content in respective wells are shown. Error bars represent the standard deviation based on three replicate experiments. B) Concentration-response curve for growth inhibition in HT29 and HCT116 colorectal carcinoma cells. Proliferation was quantified 96 hours post compound addition using a sulphorhodamine B based colorimetric assay. C) Growth inhibition following pulse-treatment of cells. Cells were treated for the time indicated after which compound containing medium was removed. Proliferation was quantified 96 hours post treatment using a sulphorhodamine B based colorimetric assay. GI50 calculations were performed by non-linear regression using the Prism V4.0 software. (TIF) [file pone.0028568.s002.tif]

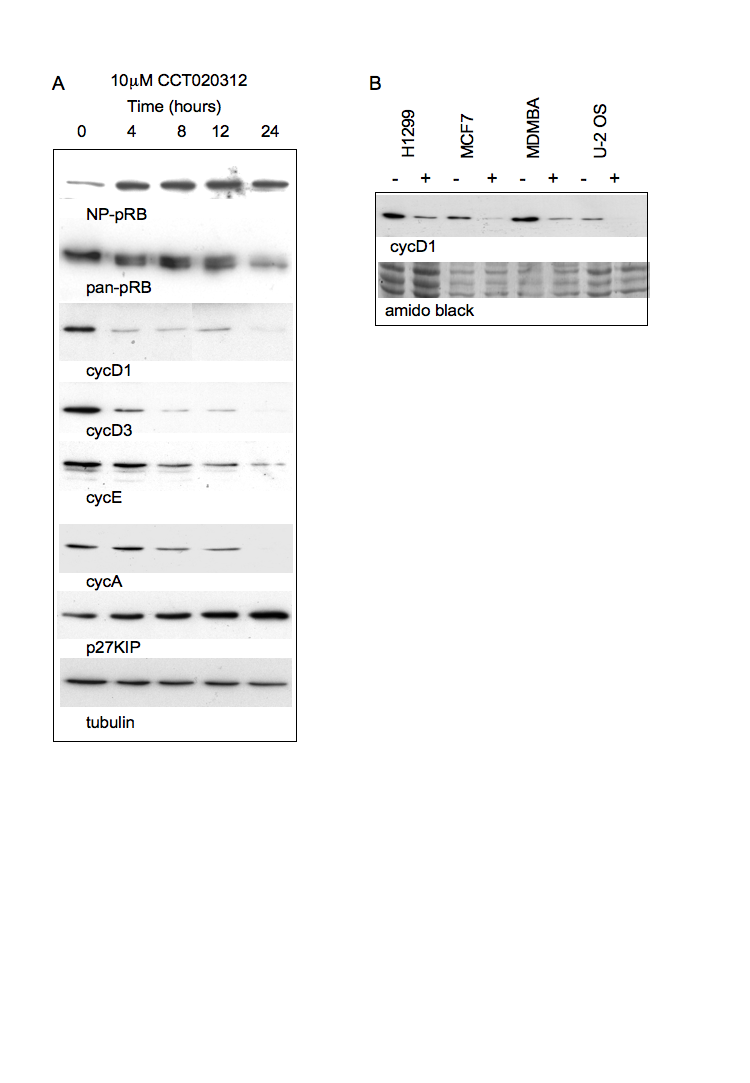

Supplement: Figure S3 — Effects of CCT020312 on expression of G1/S CDKs and their regulators. A) Changes in biomarker expression over 24 h. HT29 cells were treated with 10 µM CCT020312 for the periods indicated. Lysates were prepared at each time point and analysed as in FIG. 2E. B) Loss of cyclin D1 is widely detected in cell lines treated with CCT020312. Cell lines as indicated were treated with 10 µM CCT020312 (+) or vehicle (−) for 6 h. Lysates were analyzed for cyclin D1 expression by immunoblot. Membrane staining with amido black (A, D) or probing for tubulin (B, C) was used to reveal loading. (TIF) [file pone.0028568.s003.tif]

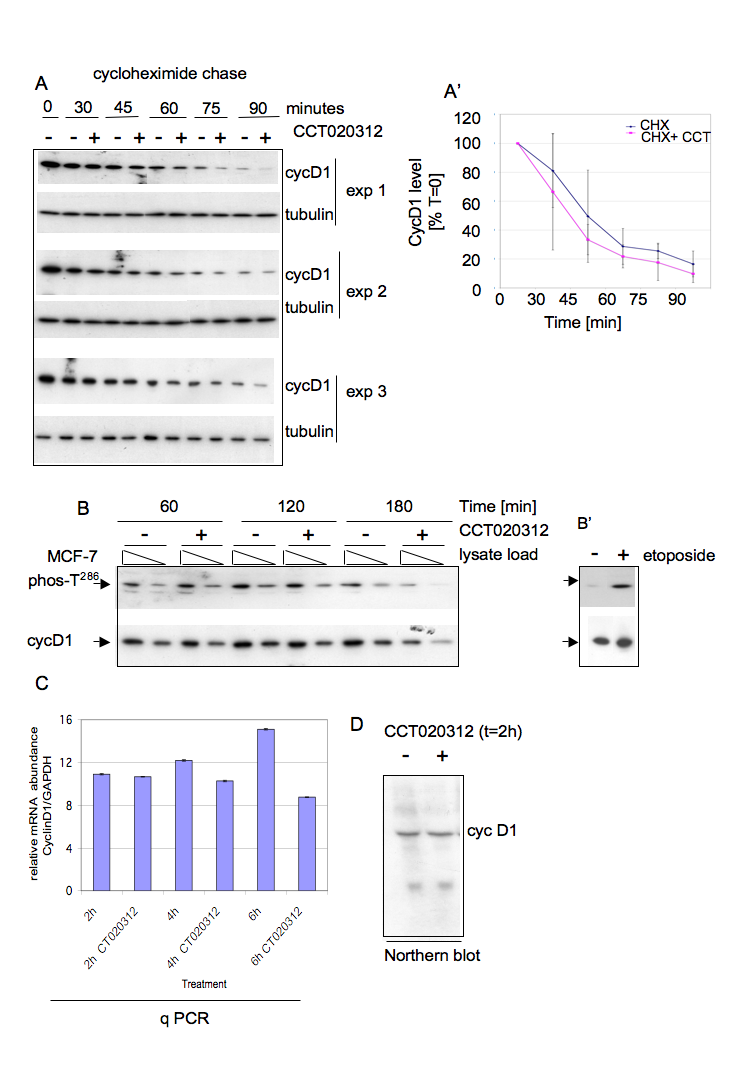

Supplement: Figure S4 — Effect of CCT020312 on cyclin D1 turnover and mRNA accumulation. A) CCT020312 does not affect Cyclin D1 stability. MCF-7 were treated with 20 µg/ml cycloheximide in combination with 10 µM CCT020312, as indicated. Lysates were analysed by Immunoblotting using anti Cyclin D1 and tubulin. A′) quantification of Cyclin D decay using cy-5 conjugated secondary antibody. Signals were quantified by PhosphoImager. B) CCT020312 does not trigger Thr286 phosphorylation of Cyclin D1. MCF-7 cells were treated with 10 µM CCT020312 (+) for the times indicated. Lysates were adjusted for protein content and analysed undiluted or diluted 1∶2 by immunoblotting blotting using antibodies as indicated. MCF-7 cells were treated with 100 µM etoposide a known inducer of cyclin D-Thr286 phosphorylation (B). C), D) Effect of CCT020312 on cyclin D1 steady mRNA state levels. MCF-7 cells were treated with 10 µM CCT020312 for the time indicated. RNA was analysed by SYBR green assisted q PCR (C), or Northern blot (D). Error bars in C represent the means of four parallel technical replicates. qPCR reactions were evaluated using the standard curve method. GAPDH was used as a reference for normalization. (TIF) [file pone.0028568.s004.tif]

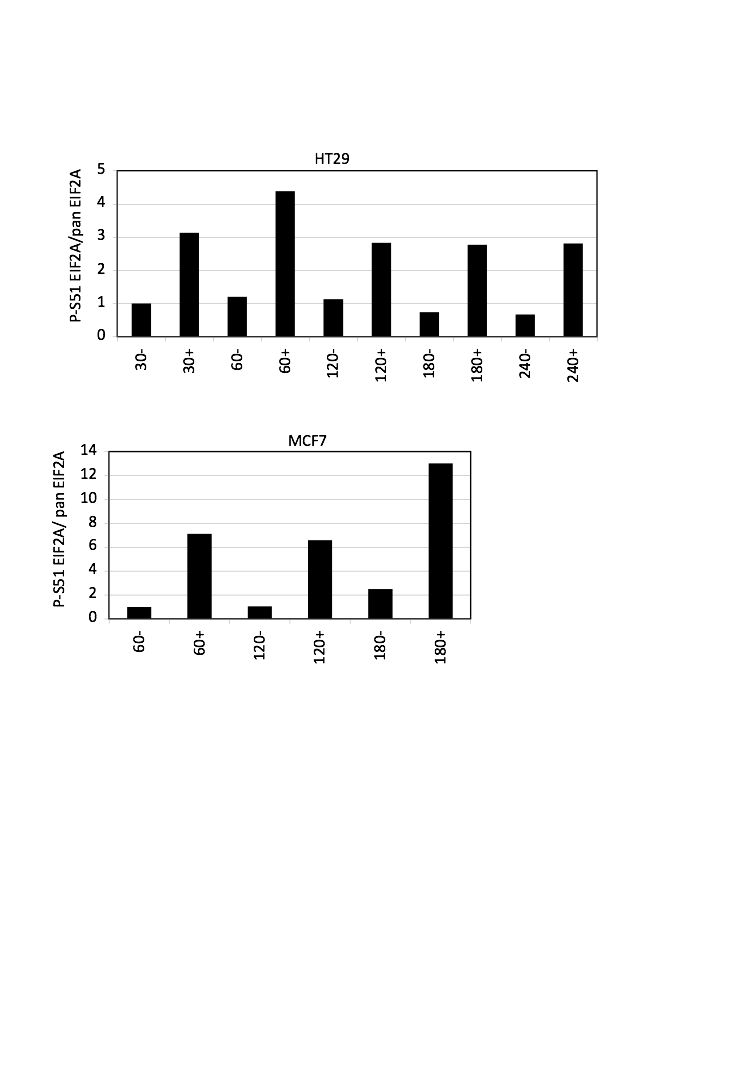

Supplement: Figure S5 — EIF2A phosphorylation following CCT020312 treatment. Signal quantification for results shown in Figure 4A. Electronic scans were produced from primary autoradiograms and analysed using ImageJ (http://rsbweb.nih.gov/ij/). Charts depict background corrected signal quantities for P-S51 EIF2A relative to the signal quantities for pan EIF2A in the same samples. (TIF) [file pone.0028568.s005.tif]

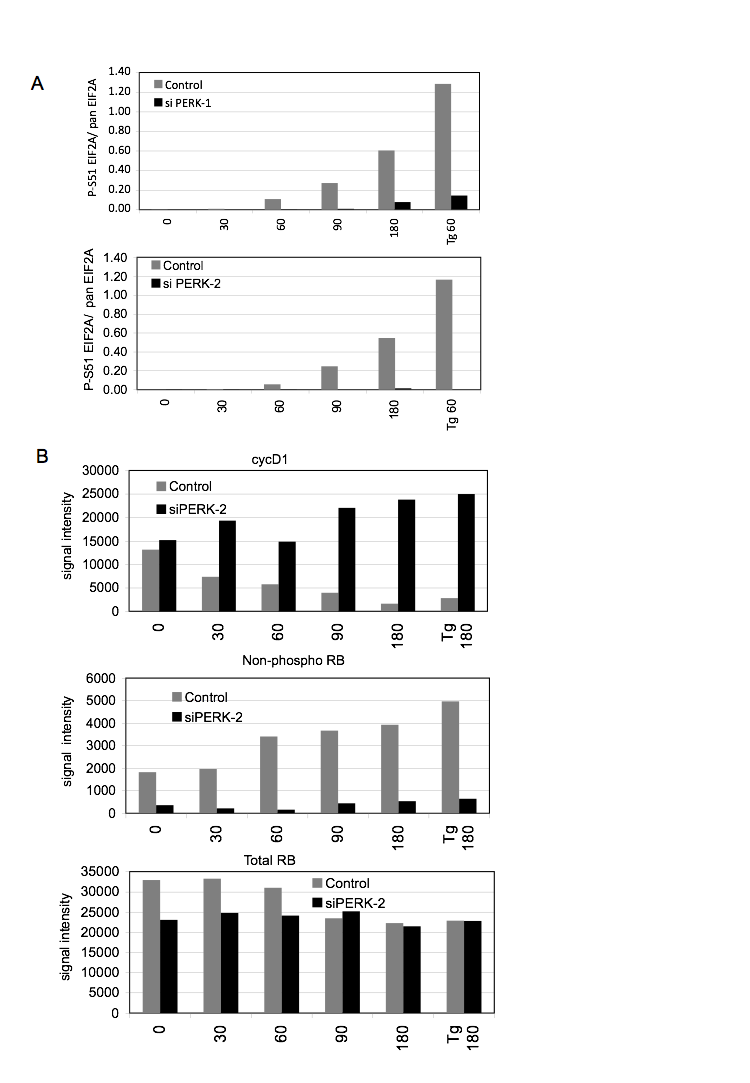

Supplement: Figure S6 — Effect of EIF2AK3/PERK ablation on CCT020312-mediated EIF2A phosphorylation and loss of cyclin D and pRB phosphorylation. A) Signal quantification for results shown in Figure 5B . Charts depict background corrected signal for P-S51 EIF2A relative to that of pan EIF2A in the same samples. B) Signal quantification for results shown in Figure 5C . Charts depict background corrected raw signal quantities for Cyc D1, nonphosphorylated RB and pan RB. Quantification was performed using electronic scans produced from primary autoradiograms. Data were analysed using ImageJ (http://rsbweb.nih.gov/ij/). (TIF) [file pone.0028568.s006.tif]

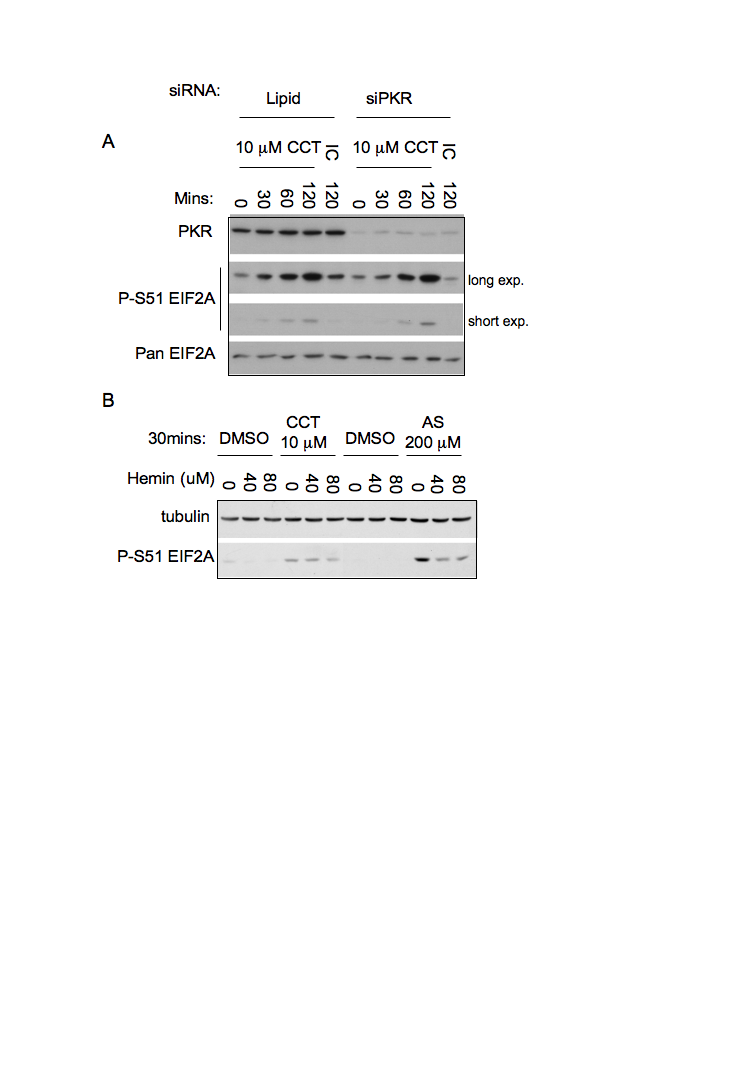

Supplement: Figure S7 — Impact of EIF2AK inhibition on CCT020312 signalling. A) EIF2AK2/PKR ablation fails to affect CCT020312 mediated EIF2A phosphorylation. U2-OS human osteosarcoma cells were transfected with 20nM siRNA targeting PKR, or a control. Following 72 hours of RNAi cells were treated with 10 µM CCT020312 or DMSO for the indicated times, or with 500 µg/ml Poly (I∶C). Cells were harvested and lysates analysed by immunoblotting using antibodies as indicated. B) EIF2AK1/HRI inhibition fails to inhibit CCT020312-mediated EIF2A phosphorylation. U2-OS cells were treated with 0, 40 or 80 µm of the EIF2AK1/HRI inhibitor hemin for 1 h. Hemin-containing media was replaced with media containing 10 µM CCT020312 or 200 µM sodium arsenide (AS) or DMSO. Cells were lysed after 30 min and lysates analysed by immunoblotting as indicated. (B) Regulation of genes responsive to integral stress response signalling. mRNA abundance for CHOP/GADD153 and WARS, previously shown to depend on stress signalling involving EIF2A phoshorylation, as determined from cDNA data on thapsigargin and CCT020312 treated cells. Expression values shown are relative to DMSO treated control samples. mRNA expression data were extracted from the microarray dataset. Normalized data are expressed as a ratio of treated cells over untreated control cells. C) Activation of EIF2A but not UPR selective signalling in mouse embryo fibroblasts (MEFs). Primary MEFs were treated with 10 µM CCT020312 or 2 µM thapsigargin for 8 hours and analysed by immunoblot using antibodies to detect markers of EIF2AK (ATF4 and CHOP) and IRE involving (XBP1s) signalling. (TIF) [file pone.0028568.s007.tif]

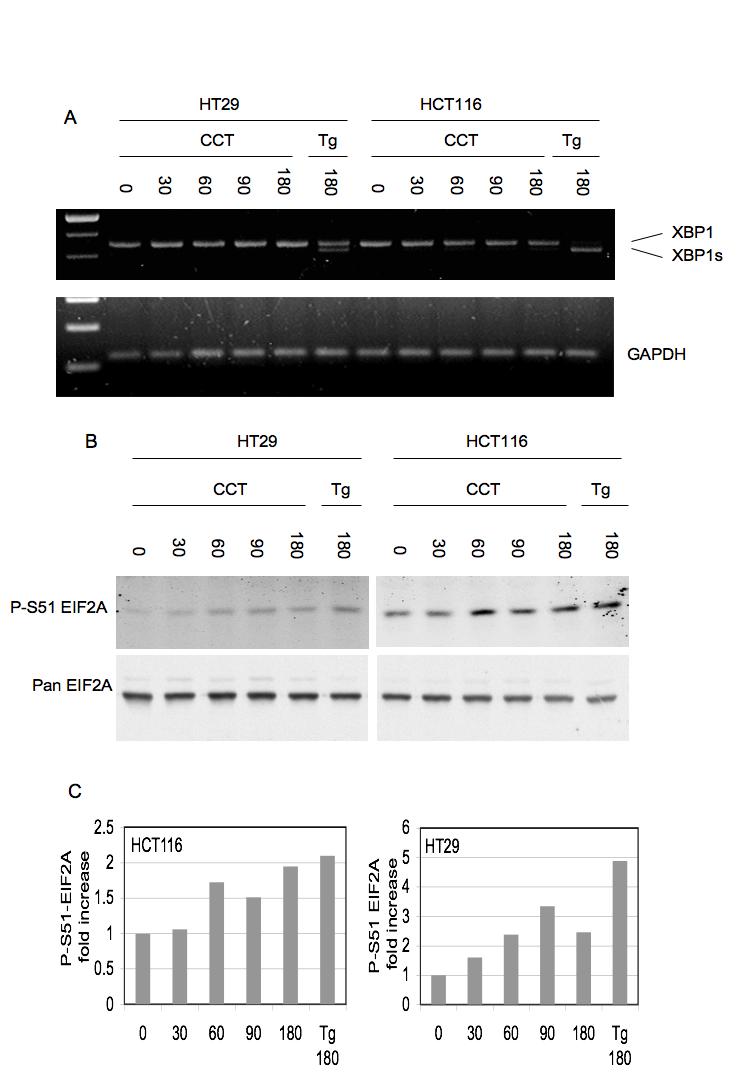

Supplement: Figure S8 — Absence of detectable XBP1 splicing in CCT020312 treated human cancer cells. A) PCR based detection of XBP1 splicing. HT29 or HCT116 cells were treated with 10 µM CCT020312 (CCT) or 2 µM thapsigargin (Tg) for the time indicated. Alternative splicing of XBP1 was detected in total RNA extracts from these cells by reverse transcription PCR. B) Detection of EIF2A phosphorylation. Extracts for protein analysis were generated in parallel to RNA preparations analysed for A. C) Signal quantification for results shown in B). Charts depict background corrected P-S51 EIF2A after normalization against pan EIF2A. Signals were quantified as for supplemental Figure 5 using Image J. (TIF) [file pone.0028568.s008.tif]

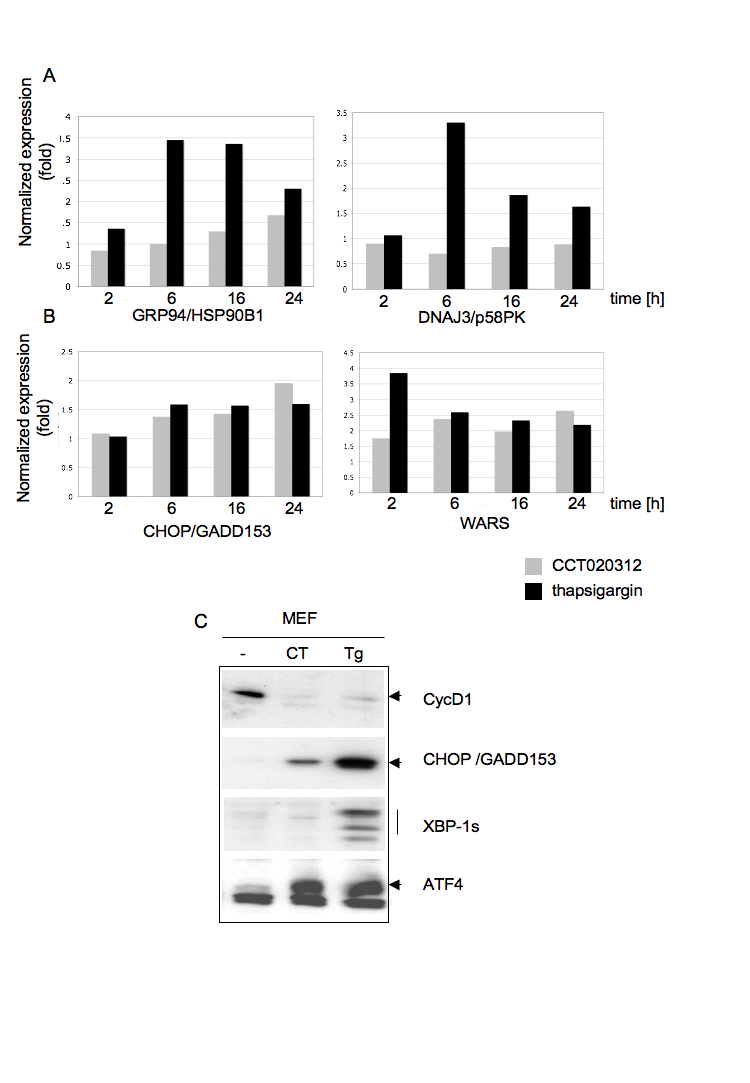

Supplement: Figure S9 — CCT020312 fails to trigger unfolded protein response selective signalling. A) Regulation of chaperone encoding genes activated downstream of XBP1. mRNA abundance for GRP94/HSP90B1 and DNAJ3/p58PK, previously shown to rely on XBP1 signalling [7] as determined from cDNA microarray data on thapsigargin and CCT020312 treated cells. Expression values shown are relative to DMSO treated control samples. mRNA expression data were extracted from the microarray dataset. Normalized data are expressed as a ratio of treated cells over untreated control cells. (B) Regulation of genes responsive to integral stress response signalling. mRNA abundance for CHOP/GADD153 and WARS, previously shown to depend on stress signalling involving EIF2A phoshorylation [8], as determined from cDNA data on thapsigargin and CCT020312 treated cells. Expression values shown are relative to DMSO treated control samples. mRNA expression data were extracted from the microarray dataset. Normalized data are expressed as a ratio of treated cells over untreated control cells. C) Activation of EIF2A but not UPR selective signalling in mouse embryo fibroblasts (MEFs). Primary MEFs were treated with 10 µM CCT020312 or 2 µM thapsigargin for 8 hours and analysed by immunoblot using antibodies to detect markers of EIF2AK (ATF4 and CHOP) and IRE involving (XBP1s) signalling. References. 7. Lee AH, Iwakoshi NN, Glimcher LH (2003) XBP-1 regulates a subset of endoplasmic reticulum resident chaperone genes in the unfolded protein response. Mol Cell Biol 23: 7448–7459. 8. Mori K (2009) Signalling pathways in the unfolded protein response: development from yeast to mammals. J Biochem 146: 743–750. (TIF) [file pone.0028568.s009.tif]

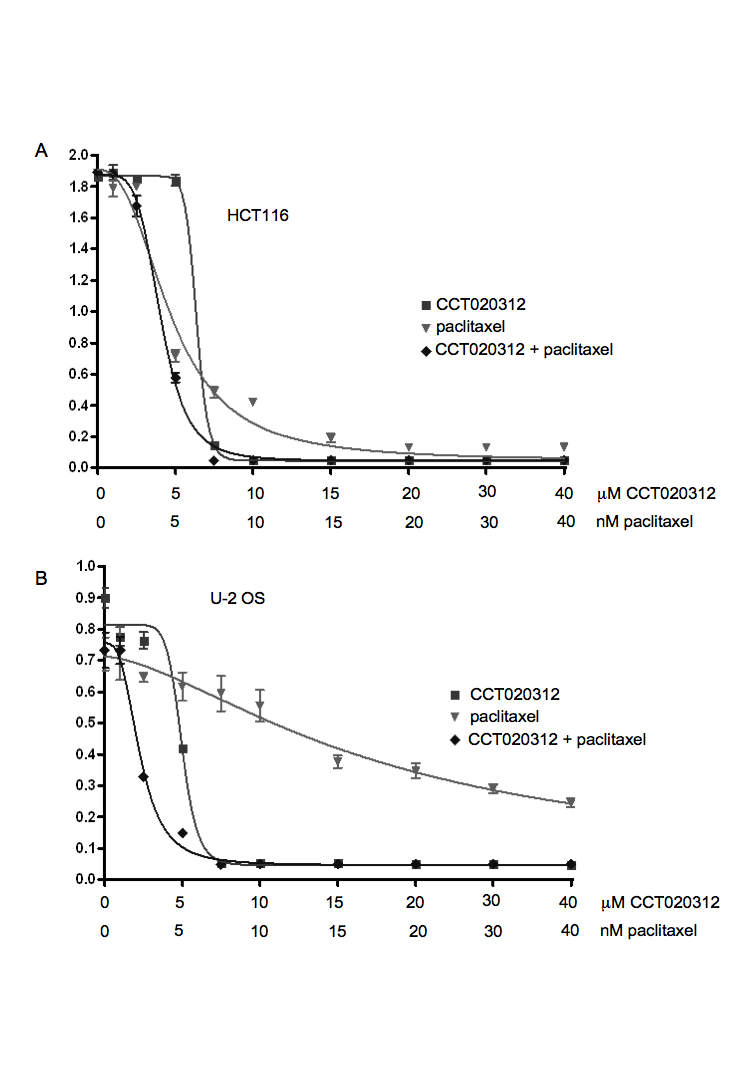

Supplement: Figure S10 — Interaction of CCT020312 and paclitaxel. A, B) Cell proliferation activity of cells following treatment with paclitaxel, CCT020312 or the combination thereof, as determined using a 96 hour sulphorhodamine B (SRB) colorimetric assay run in a 96-well assay format. Agent concentrations were as indicated. Data were used to calculate combination indices for Figure 8C. (TIF) [file pone.0028568.s010.tif]
