# Supplementary material for: Mechanism-Based Screen for G1/S Checkpoint Activators Identifies a Selective Activator of EIF2AK3/PERK Signalling
Source: PLoS One. 2012 Jan 12;7(1):e28568. doi: 10.1371/journal.pone.0028568 (PMC3257223; doi:10.1371/journal.pone.0028568)
Supplement: Table S1 — Prediction of small molecule agents connected to the mechanism of action of CCT020312. The top ten hits are shown. Analysis output based on cmap 02 (http://www.broadinstitute.org/cmap/http://www.broadinstitute.org/cmap/). (DOC) [file pone.0028568.s011.doc]

Table S1

| **rank** | **Name (compound)** | **P value** | **specificity** | **enrichment** |
| --- | --- | --- | --- | --- |
| 1 | thioridazine | 0 | 0.0731 | 0.644 |
| 2 | 15-delta prostaglandin J2 | 0 | 0.1062 | 0.628 |
| 3 | trichostatin A | 0 | 0.2796 | 0.508 |
| 4 | MG-262 | 0.00008 | 0.0703 | 0.961 |
| 5 | H-7 | 0.00014 | 0.0985 | -0.906 |
| 6 | geldanamycin | 0.00022 | 0.1467 | 0.534 |
| 7 | withaferin A | 0.00044 | 0.0842 | 0.865 |
| 8 | parthenolide | 0.00072 | 0.1258 | 0.851 |
| 9 | flunixin | 0.00088 | 0 | -0.783 |
| 10 | hydrocotarnine | 0.00107 | 0.011 | -0.842 |
