# Supplementary material for: Mechanism-Based Screen for G1/S Checkpoint Activators Identifies a Selective Activator of EIF2AK3/PERK Signalling
Source: PLoS One. 2012 Jan 12;7(1):e28568. doi: 10.1371/journal.pone.0028568 (PMC3257223; doi:10.1371/journal.pone.0028568)
Supplement: Results S1 — Assessment of hit matter. (DOC) [file pone.0028568.s012.doc]

**SUPPLEMENT TO RESULTS**

**Assessment of hit matter**

Two of the confirmed hits, CCT001959 and CCT005616, are lipophilic compounds with a planar aromatic core and an associated propensity to intercalate DNA and bind in a promiscuous manner to multiple protein targets yielding broad secondary pharmacology and associated *in vivo* toxicity risks . CCT011668 contains an aminothiazole core scaffold with an associated risk of adverse toxicology and secondary pharmacology through oxidative metabolism . Broad secondary pharmacology and associated non-specific mode of action of these compounds, coupled with poor physicochemical properties, renders them unattractive as chemical tools for studying biological pathways and the identification of biochemical targets . Three other compounds (CCT004005, CCT009263 and CCT008234) are quaternary salts with undefined counterions. Quaternary salts have poor cell permeability consistent with the reduced potency of CCT009263 and CCT008234. The remaining two compounds, CCT020312 and CCT039836, have good potency, however, CCT039836, the most potent compound, has an uncommon chemical structure. The CCT039836 core scaffold is not a classic heteroaromatic ring system and could be a source of chemical instability. CCT020312 is an acylated dihydropyrazole where oxidative instability to yield the aromatic pyrazole core is possible. In addition, CCT020312 is a lipophilic base indicating a potential for polypharmacology, although the substitution pattern of the compound offers scope for the discovery of a less lipophilic, achiral pharmacophore.

**References**

1. Hughes JD, Blagg J, Price DA, Bailey S, Decrescenzo GA, et al. (2008) Physiochemical drug properties associated with in vivo toxicological outcomes. Bioorg Med Chem Lett 18: 4872-4875.

2. Blagg J (2006) Structure-activity relationships for in vitro and in vivo toxicity. . Annual Reports in Medicinal Chemistry 41: 353-368.

3. Workman P, Collins I (2011) Probing the probes: fitness factors for small molecule tools. Chem Biol 17: 561-577.

4. Stahl PH, Nakano M (2008) Pharmaceutical salts: Properties, Selection and Use. Verlag Helvetica Chimica Acta ISBN-10 3-906390-58-6.: 86-89.
