# Supplementary material for: Mechanism-Based Screen for G1/S Checkpoint Activators Identifies a Selective Activator of EIF2AK3/PERK Signalling
Source: PLoS One. 2012 Jan 12;7(1):e28568. doi: 10.1371/journal.pone.0028568 (PMC3257223; doi:10.1371/journal.pone.0028568)
Supplement: Methods and Materials S1 — XBP1 splicing and General Methods for the preparation of compounds. (DOC) [file pone.0028568.s013.doc]

**SUPPLEMENT TO METHODS AND MATERIALS**

*XBP1 splicing assay.*

HT29 or HCT116 cells were seeded to 6-well tissue culture plates (4x10^5^ or 2x10^5^ per well, respectively) and grown for 48 hours. Duplicate plates were then treated with compound for set times and processed for either protein or RNA analysis. RNA was isolated using Qiagen’s RNeasy mini kit using the QIA shredder option. RNA samples were quantified by spectrophotometry. First strand cDNA synthesis was performed using 1 μg of RNA sample in a 20 μl reaction containing 0.5 mM dNTPs, 3.45 μM random hexamer primers, 200 units of reverse transcriptase, 1 unit of RNAse inhibitor (Sigma R2520). Reactions proceeded for a single thermal cycle of 10 minutes at room temperature, 50 minutes at 37°C and then 10 minutes at 88°C. 3 μl of the resulting cDNA samples were then used in 50 μl PCR reactions to detect XBP1 and XBP1s splicing products as described in using primers 5’-CCTTGTAGTTGAGAACCAGG-3’ (forward) and 5’-GGGGCTTGGTATATATGTGG-3 (reverse). PCR reactions contained 100nM primers, 200 μM dNTP, 1 unit of platinum Taq DNA polymerase (Invitrogen) and 1.5 mM MgCl_2_. Reactions were placed at 94°C for 2 minutes, then subjected to 30 cycles of 94°C for 15 seconds, 60°C for 1 minute, and 72°C for 30 seconds. PCR products were analysed on 2% agarose gels containing ethidium bromide. Parallel PCR reactions for GAPDH were run to confirm adequate template input.

*General Methods for the preparation of compounds*

Compounds 1 to 11: All anhydrous solvents and reagents were obtained from commercial suppliers and used without any further purification unless otherwise noted. ^1^H-nuclear magnetic resonance spectra were recorded on a Bruker 250MHz Avance instrument or on a Bruker Ultrashield 500MHz Avance using an internal deuterium lock. Chemical shifts were measured in parts per million (ppm) relative to tetramethylsilane (δ = 0) using the following internal references for residual protons in the solvent: CDCl_3_ (δ 7.26) and DMSO-*d6* (δ 2.50). Multiplicities are indicated by s (singlet), d (doublet), t (triplet), q (quartet), m (multiplet), br (broad) or combinations thereof. ^13^C-nuclear magnetic resonance spectra were routinely recorded at 62.5MHz on the Bruker 250MHz spectrometer using an internal deuterium lock. All chemical shift values were reported in ppm relative to tetramethylsilane (δ = 0). The following internal references were used: CDCl_3_ (δ 77.0) and DMSO-*d6* (δ 39.5). Flash column chromatography was carried out on Merck silica gel 60 (0.015-0.040 mm) employing mixture of specified solvent as eluent. Thin-layer chromatography (TLC) was performed on Merck silica gel (Merck 60 PF_254_) plates. Silica TLC plates were visualized under UV light. Melting points were determined on a Leica Gallen III melting point apparatus and are uncorrected. LC-MS analyses were performed on a Micromass LCT / Water’s Alliance 2795 HPLC system with a Discovery column from Supelco at a temperature of 22^o^C. UV detection was at 254nm and ionisation was positive or negative ion electrospray. Molecular weight scan range was 150‑1000. Samples were supplied as 1 mg/ml in methanol or methanol/DMSO with 3 μl injected on a partial loop fill.

*Preparation of CCT020312*


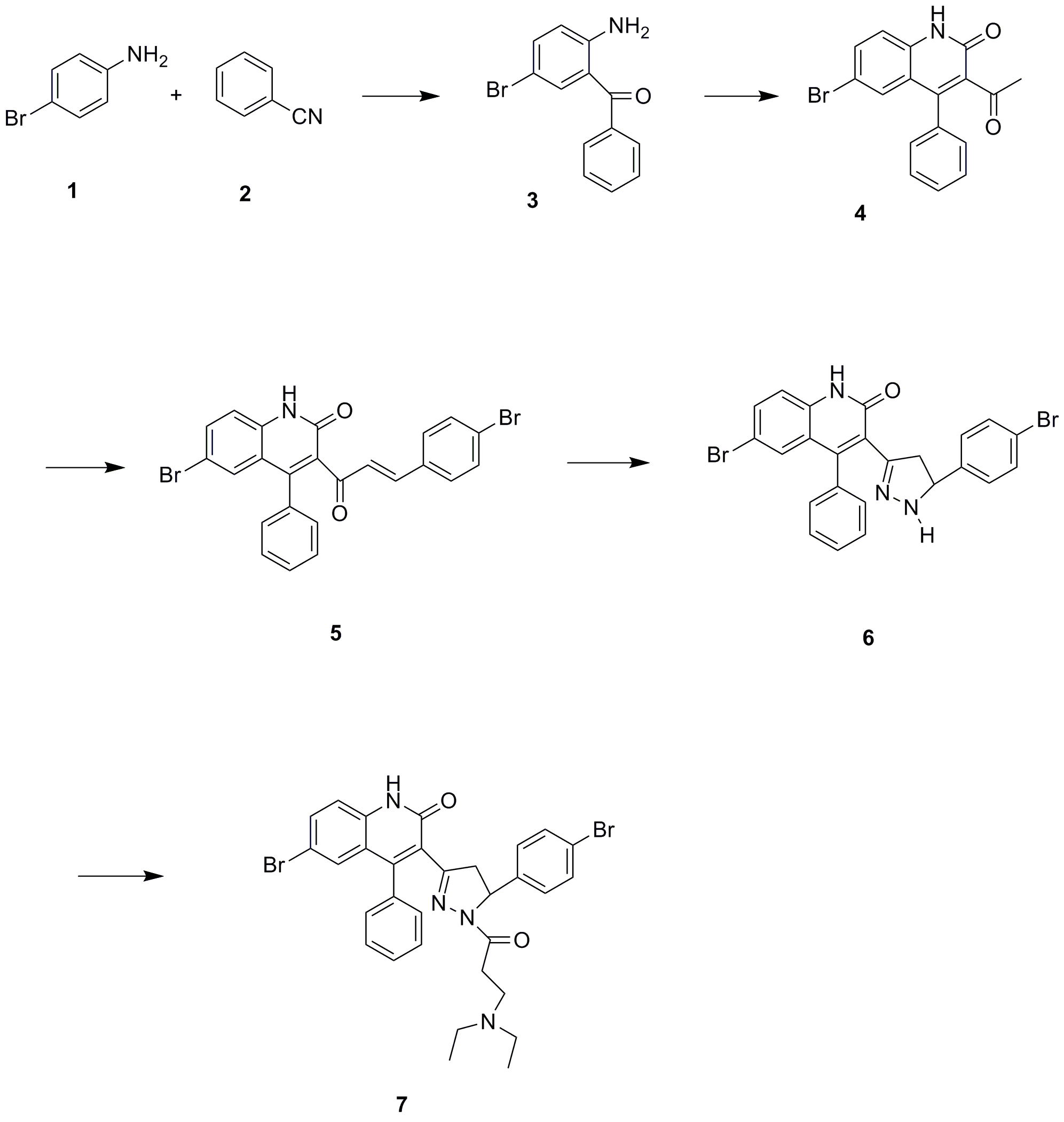


**Scheme 1**

**(2-Amino-5-bromo-phenyl)-phenyl-methanone (3)**

To a stirred solution of boron trichloride (1M in heptane, 28 mL, 1.3 eq, 28.31 mmol) in chlorobenzene (100 mL) at 0°C under an argon atmosphere was added 4-bromoaniline (**1**, 7.51 g, 2 eq, 43.66 mmol) followed by benzonitrile (**2**, 2.04 mL, 1 eq, 21.78 mmol) and gallium chloride (5g, 1.3 eq, 28.31 mmol). The reaction mixture was stirred at RT for 1 h and then placed in a preheated oil bath at 100°C and stirred at this temperature for 20 h. The reaction mixture was allowed to cool to room temperature and HCl (4N, 100 mL) was added and stirred at 100°C for 30 min. The mixture was partitioned with CHCl_3_ (2 x 250 mL) and the organic phase washed with brine (50 mL), dried (MgSO_4_) and evaporated *in vacuo*. The residue was extracted into ether/ hexane (300 mL, 1:1), filtered, and the filtrate evaporated *in vacuo.* The resulting solid was triturated with hexane (100 mL), filtered, and the filtrate evaporated *in vacuo* to afford 2-amino-5-bromo-phenyl)-phenyl-methanone (**3**) as a beige solid; Yield: 1.99 g (33%). mp 108-109°C.  ^1^H-NMR (CDCl_3_, 250 MHz): δ 6.10 (bs, 2H, -NH_2_), 6.67 (d, 1H, J = 8.77 Hz, Bz-H), 7.39 (dd, 1H, J = 2.25 + 11.14 Hz, Bz-H), 7.51 (m, 1H, Bz-H), 7.54 (m, 1H, Bz-H), 7.58 (m, 2H, 2 x Bz-H), 7.65 (m, 1H, Bz-H), 7.68 (m, 1H, Bz-H). ^13^C-NMR (CDCl_3_, 62.5 MHz): δ 106.7, 112.2, 118.8, 119.5, 128.4, 129.1, 131.6, 136.3, 136.9, 143.2, 179.5.

**3-Acetyl-6-bromo-4-phenyl-1H-quinolin-2-one (4)**

A stirred solution 2-amino-5-bromo-phenyl)-phenyl-methanone (**3,** 1.95 g, 1 eq, 7.01 mmol) in excess ethyl acetoacetate (7 mL), degassed under argon, was heated at 185°C for 16h. The reaction mixture was allowed to cool to room temperature and the product precipitated with ether (20 mL), filtered, and the residue dried *in vacuo* to afford 3-acetyl-6-bromo-4-phenyl-1H-quinolin-2-one (**4**) as a beige solid; Yield: 1.08 g (46%). mp 298-300°C.  ^1^H-NMR (CDCl_3_, 250 MHz): δ 2.30 (s, 3H, -CH_3_), 7.30 (s,1H, Bz-H), 7.36 (m, 2H, 2 x Bz-H), 7.42 (d, 1 H, J = 2.07 Hz, Bz-H), 7.55 (m, 3H, 3 x Bz-H), 7.67 (dd, 1H, J = 2.13 + 8.71 Hz), 12.49 (bs, 1H, -NH). ^13^C-NMR (CDCl_3_, 62.5 MHz): δ 31.6, 116.0, 118.2, 121.4, 128.9, 129.00, 129.4, 129.9, 133.4, 134.5, 137.0, 147.9, 163.5, 179.6, 201.5.

**6-Bromo-3-[3-(4-bromo-phenyl)-acryloyl]-4-phenyl-1H-quinolin-2-one (5)**

To a stirred solution of 3-acetyl-6-bromo-4-phenyl-1H-quinolin-2-one (**4,** 0.92 g, 1 eq, 2.69 mmol) in ethanol (25 mL), was added 4-bromobenzaldehyde (0.75 g, 1.5 eq, 4.04 mmol), followed by a solution of KOH (0.30 g, 2 eq, 5.36 mmol) in water (25 mL) and was heated at 65°C for 16h. The solvent was evaporated *in vacuo*, and the residue partitioned between DCM (1 L) and water (500mL). The organic phase was washed with brine (50 mL), dried (MgSO_4_) and evaporated *in vacuo.* The residue was triturated with ether (2 x 20 mL), filtered, and the residue dried *in vacuo* to afford 6-bromo-3-[3-(4-bromo-phenyl)-acryloyl]-4-phenyl-1H-quinolin-2-one (**5**) as a white solid; Yield: 0.90 g (66%). mp 241-243°C.  ^1^H-NMR (CDCl_3_, 250 MHz): δ 6.69 (s, 1H, C=CH) 6.76 (s, 1H, C=CH), 7.32 (m, 7H, 7 x Bz-H), 7.47 (m, 5H, 5 x Bz-H), 7.52 (s, 1H, Bz-H), 7.60 (dd, 1H, Bz-H), 12.38 (bs, 1H, -NH).^13^C-NMR (CDCl_3_, 62.5 MHz): δ 116.05, 118.40, 121.39, 125.20, 127.59, 128.77, 129.06, 129.35, 129.78, 131.15, 131.35, 131.65, 132.19, 133.17, 133.45, 134.42, 137.27, 144.16, 149.55, 161.61, 193.10.

**6-Bromo-3-[5-(4-bromo-phenyl)-4,5-dihydro-1H-pyrazol-3-yl]-4-phenyl-1H-quinolin-2-one (6)**

A stirred solution 6-bromo-3-[3-(4-bromo-phenyl)-acryloyl]-4-phenyl-1H-quinolin-2-one (**5,** 0.80 g, 1 eq, 1.73 mmol) in EtOH (50 mL) and hydrazine hydrate (0.188 mL, 3.5 eq, 6.05 mmol) under an argon atmosphere, was heated at 95°C for 16h. The solvent was evaporated *in vacuo*, and the residue partitioned between DCM (1 L) and water (500mL). The organic phase was dried (MgSO_4_) and evaporated *in vacuo* to afford 6-bromo-3-[5-(4-bromo-phenyl)-4,5-dihydro-1H-pyrazol-3-yl]-4-phenyl-1H-quinolin-2-one (**6**) as a yellow solid; Yield: 0.90 g (99%) which was used directly for the preparation of **7**.

**6-Bromo-3-[5-(4-bromo-phenyl)-1-(3-diethylamino-propionyl)-4,5-dihydro-1H-pyrazol-3-yl]-4-phenyl-1H-quinolin-2-one (7) CCT020312.**

A solution of 6-bromo-3-[5-(4-bromo-phenyl)-4,5-dihydro-1H-pyrazol-3-yl]-4-phenyl-1H-quinolin-2-one (**6,** 0.88 g, 1 eq, 1.69 mmol), diethylaminopropionic acid (338 mg, 1.1 eq, 1.86 mmol) and 1,3-diisopropylcarbodiimide (0.34 mL, 1.3 eq, 2.20 mmol) in DCM/ DMA (60 mL, 2:1) was stirred under an argon atmosphere at RT for 16h. The solvent was evaporated *in vacuo*, and the residue partitioned between DCM (250 mL) and water (250mL). The organic phase was washed with brine (50 mL), dried (MgSO_4_) and evaporated *in vacuo.* The residue was purified by column chromatography on silica gel eluted with DCM: MeOH (90:10) to afford 6-bromo-3-[5-(4-bromo-phenyl)-1-(3-diethylamino-propionyl)-4,5-dihydro-1H-pyrazol-3-yl]-4-phenyl-1H-quinolin-2-one (**7**) as a yellow solid; Yield: 0.82 g (75%). mp 166-170°C.  ^1^H-NMR (d_6_-DMSO, 500 MHz): δ 1.34-0.89 (6H, t, J = 7.25Hz); 2.21-2.29 (1H, m); 2.38 (4H, q, J = 6.94Hz); 2.40-2.47 (1H, m); 2.53, (2H, br m); 2.80 (1H, dd, J = 4.73, 18.6Hz); 3.74 (1H, dd, J = 11.98, 18.3Hz); 5.31 (1H, dd, J = 4.41, 11.98Hz); 6.78 (2H, d, J = 8.20Hz); 7.07 (1H, d, J = 2.21Hz); 7.27 (1H, d, br, J = 8.20Hz); 7.39 (2H, d, J = 8.83Hz); 7.41 (2H, d, J = 8.51Hz); 7.48-7.53 (1H, m); 7.53-7.56 (2H, m); 7.73 (1H, dd, J = 2.21, 8.83Hz); 12.3 (1H, br s, NH). ^13^C-NMR (CDCl_3_, 125MHz): δ 11.86, 31.10, 45.23, 46.79, 47.74, 59.15, 116.05, 117.71, 121.37, 121.98, 124.02, 127.77, 128.49, 128.63, 128.84, 128.91, 129.26, 130.31, 131.79, 134.53, 134.66, 136.99, 140.84, 151.89, 162.46. Mass Spec: Found 648.07211, C_31_H_30_^79^Br_2_N_4_O_2_  requires 648.07355.

*Synthesis of CCT020312 analogues*


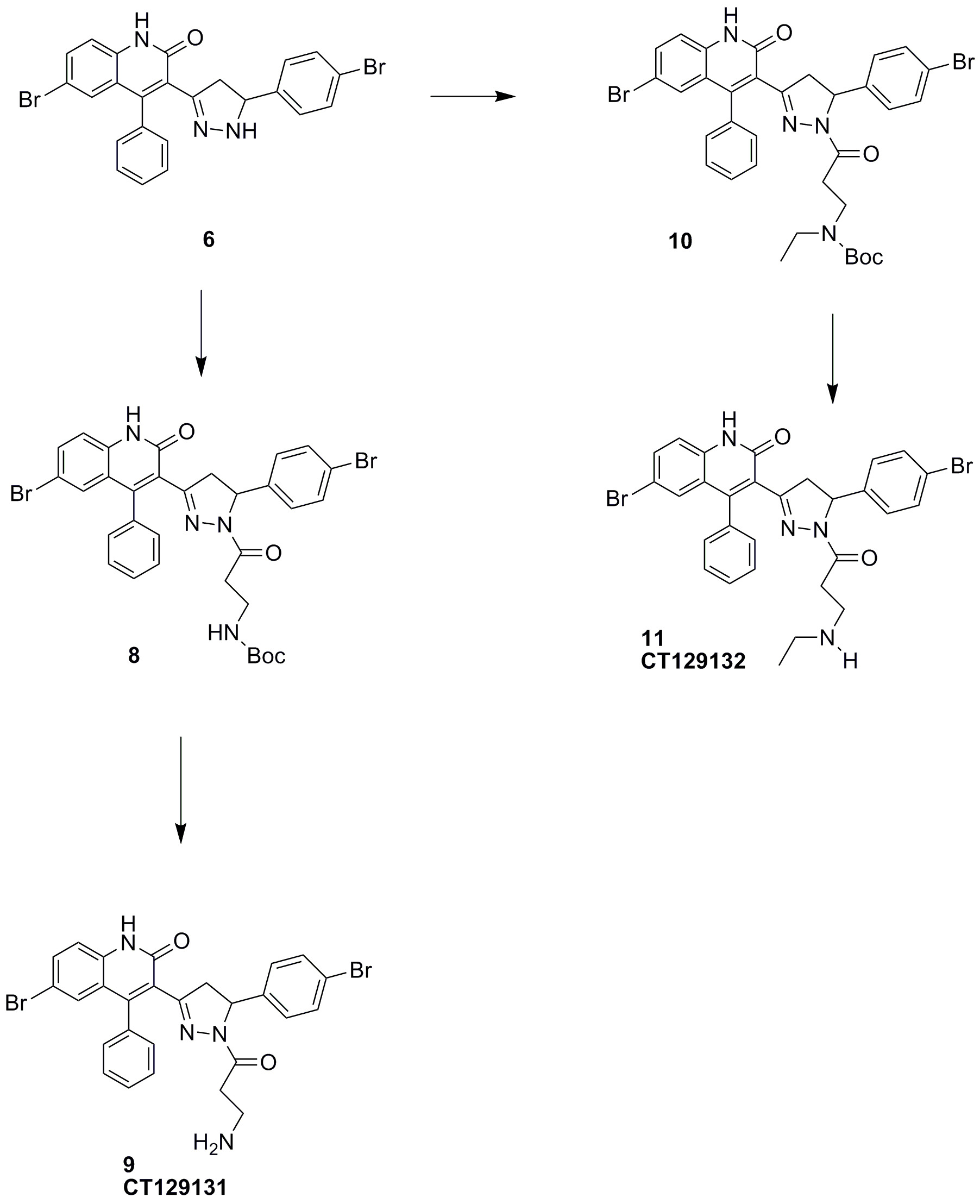


**Scheme 2**

**{3-[3-(6-Bromo-2-oxo-4-phenyl-1,2-dihydro-quinolin-3-yl)-5-(4-bromo-phenyl)-4,5-dihydro-pyrazol-1-yl]-3-oxo-propyl}-carbamic acid tert-butyl ester (8)**

A stirred solution 6-bromo-3-[5-(4-bromo-phenyl)-4,5-dihydro-1H-pyrazol-3-yl]-4-phenyl-1H-quinolin-2-one (**6**, 20 mg, 1 eq, 0.04 mmol), 3-(tert-butoxycarbonylamino)propanoic acid (16 mg, 2.2 eq, 0.08 mmol) and 1,3-diisopropylcarbodiimide (0.014 mL, 2.3 eq, 0.09 mmol) in DCM/DMA (2.5 mL, 4:1) under an argon atmosphere, was stirred at RT for 16h. The solvent was evaporated *in vacuo*, and the residue was purified by preparative TLC eluted with DCM: MeOH (90:10) to afford {3-[3-(6-bromo-2-oxo-4-phenyl-1,2-dihydro-quinolin-3-yl)-5-(4-bromo-phenyl)-4,5-dihydro-pyrazol-1-yl]-3-oxo-propyl}-carbamic acid tert-butyl ester (**8**) as a yellow solid; Yield: 20 mg (75%). LCMS: single peak, M+Na^+^ 714.6484, 716.6328, 718.6490.

**3-[1-(3-Amino-propionyl)-5-(4-bromo-phenyl)-4,5-dihydro-1H-pyrazol-3-yl]-6-bromo-4-phenyl-1H-quinolin-2-one (9). CT129131**

To a stirred solution {3-[3-(6-bromo-2-oxo-4-phenyl-1,2-dihydro-quinolin-3-yl)-5-(4-bromo-phenyl)-4,5-dihydro-pyrazol-1-yl]-3-oxo-propyl}-carbamic acid tert-butyl ester (**8,** 10 mg, 1 eq, 0.014 mmol) in AcOH (2.3 mL) at RT was added a solution of HCl (conc., 0.45 mL) and dioxane (0.28 mL) and was stirred at RT for 30 min. The solvent was evaporated *in vacuo* and the residue triturated with ether (10 mL) to afford 3-[1-(3-amino-propionyl)-5-(4-bromo-phenyl)-4,5-dihydro-1H-pyrazol-3-yl]-6-bromo-4-phenyl-1H-quinolin-2-one (**9**) as a beige solid; Yield: 7 mg (70%). LCMS: single peak, M+H^+^ 593 (50.8%), 595 (100%), 597 (57.3%).

**{3-[3-(6-Bromo-2-oxo-4-phenyl-1,2-dihydro-quinolin-3-yl)-5-(4-bromo-phenyl)-4,5-dihydro-pyrazol-1-yl]-3-oxo-propyl}-ethyl-carbamic acid tert-butyl ester (10)**

A stirred solution 6-bromo-3-[5-(4-bromo-phenyl)-4,5-dihydro-1H-pyrazol-3-yl]-4-phenyl-1H-quinolin-2-one (**6**, 20 mg, 1 eq, 0.04 mmol), 3-[tert-butoxycarbonyl(ethyl)amino]propanoic acid (18 mg, 2.2 eq, 0.08 mmol) and 1,3-diisopropylcarbodiimide (0.014 mL, 2.3 eq, 0.09 mmol) in DCM/DMA (2.5 mL, 4:1) under an argon atmosphere, was stirred at RT for 16h. The solvent was evaporated *in vacuo*, and the residue was purified by preparative TLC eluted with DCM: MeOH (90:10) to afford {3-[3-(6-bromo-2-oxo-4-phenyl-1,2-dihydro-quinolin-3-yl)-5-(4-bromo-phenyl)-4,5-dihydro-pyrazol-1-yl]-3-oxo-propyl}-carbamic acid tert-butyl ester (**10**) as a yellow solid; Yield: 14 mg (50%). LCMS: single peak, M+Na^+^ 742.6793, 744.6564, 746.6688.

**6-Bromo-3-[5-(4-bromo-phenyl)-1-(3-ethylamino-propionyl)-4,5-dihydro-1H-pyrazol-3-yl]-4-phenyl-1H-quinolin-2-one (11). CT129132.**

To a stirred solution {3-[3-(6-bromo-2-oxo-4-phenyl-1,2-dihydro-quinolin-3-yl)-5-(4-bromo-phenyl)-4,5-dihydro-pyrazol-1-yl]-3-oxo-propyl}-carbamic acid tert-butyl ester (**10**, 10 mg, 1 eq, 0.014 mmol) in AcOH (2.3 mL) at RT was added a solution of HCl (conc., 0.45 mL) and dioxane (0.28 mL) and was stirred at RT for 30 min. The solvent was evaporated *in vacuo* and the residue triturated with ether (10 mL) to afford 6-bromo-3-[5-(4-bromo-phenyl)-1-(3-ethylamino-propionyl)-4,5-dihydro-1H-pyrazol-3-yl]-4-phenyl-1H-quinolin-2-one (**11**) as a beige solid; Yield: 6 mg (70%). LCMS: single peak, M+H^+^ 621 (50.0%), 623 (100%), 625 (49.1%).

Compounds 12-26: Microwave reactions were performed in a Biotage initiator. LCMS Methods: samples were analysed by High Performance Liquid Chromatography-Mass Spectrometry employed the following conditions:

Analytical LCMS method 1 employed Gilson 306 pumps, Gilson 811C mixer, Gilson 806 manometric module and Gilson UV/VIS 152 detector at 254 nm wavelength. The mass spectrometer was a Finnigan AQA and a Phenomenex Luna, 5 μm pore size, C18 column of dimensions 50 x 4.60 mm was used. The injection volume was 10 μL. The mobile phase consisted of a mixture of water and acetonitrile containing 0.1% formic acid. The eluent flow rate was 1 mL/min, using 95% water: 5% acetonitrile, changed linearly to 2% water: 98% acetonitrile over 3 min and then maintained at this mixture for 5 min.

Analytical LCMS method 2 employed Gilson 306 pumps, Gilson 811C mixer, Gilson 806 manometric module, and Gilson UV/VIS 152 detector at 254 nm wavelength. The mass spectrometer was a Finnigan AQA and a Waters SunFire, 5 μm pore size, C18 column of dimensions 50 x 4.60 mm was used. The injection volume was 10 μL. The mobile phase consisted of a mixture of water and acetonitrile containing 0.1% formic acid. The eluent flow rate was 1.5 mL/min, using 95% water: 5% acetonitrile, changed linearly to 5% water: 95% acetonitrile over 5.5 min and then maintained at this mixture for 2 min.

###### Samples purified by Prep HPLC method 1 used the following conditions:

Waters 515 pumps, a Waters 2525 mixer and a Waters 2996 diode array detector. The detection was performed between 210 nm and 650 nm. The mass spectrometer was a Waters micromass ZQ and a SunFire, 5 μm pore size, C18 column of dimensions 50 x 19 mm was used. The injection volume was up to 500 µL of solution at a maximum concentration of 50 mg/mL. The mobile phase consisted of a mixture of water and acetonitrile containing 0.1% formic acid. The eluent flow rate was 25 mL/min using 95% water, 5% acetonitrile, changing linearly over 5.3 min to 95% MeCN, 5% water, and maintaining for 0.5 min.

#### Samples purified by Prep HPLC method 3 employed the following conditions:

Gilson 306 pumps, Gilson 811C mixer, Gilson 806 manometric module, and Gilson UV/VIS 152 detector at 254 nm wavelength. Sunfire Prep C18 OBD, 5 micrometre, 19x50 mm column. Start at 12 mL/min 95% water (with 0.1% trifluoroacetic acid) 5% acetonitrile (with 0.1% trifluoroacetic acid) ramping up to 3% water (with 0.1% trifluoroacetic acid) 97% acetonitrile (with 0.1% trifluoroacetic acid) over 17 min. Hold at this eluent mixture for 7.5 min then 0.5 min back to the starting conditions equilibrating for 4 min. Peak detection limit 500, collecting 2 mL fractions.


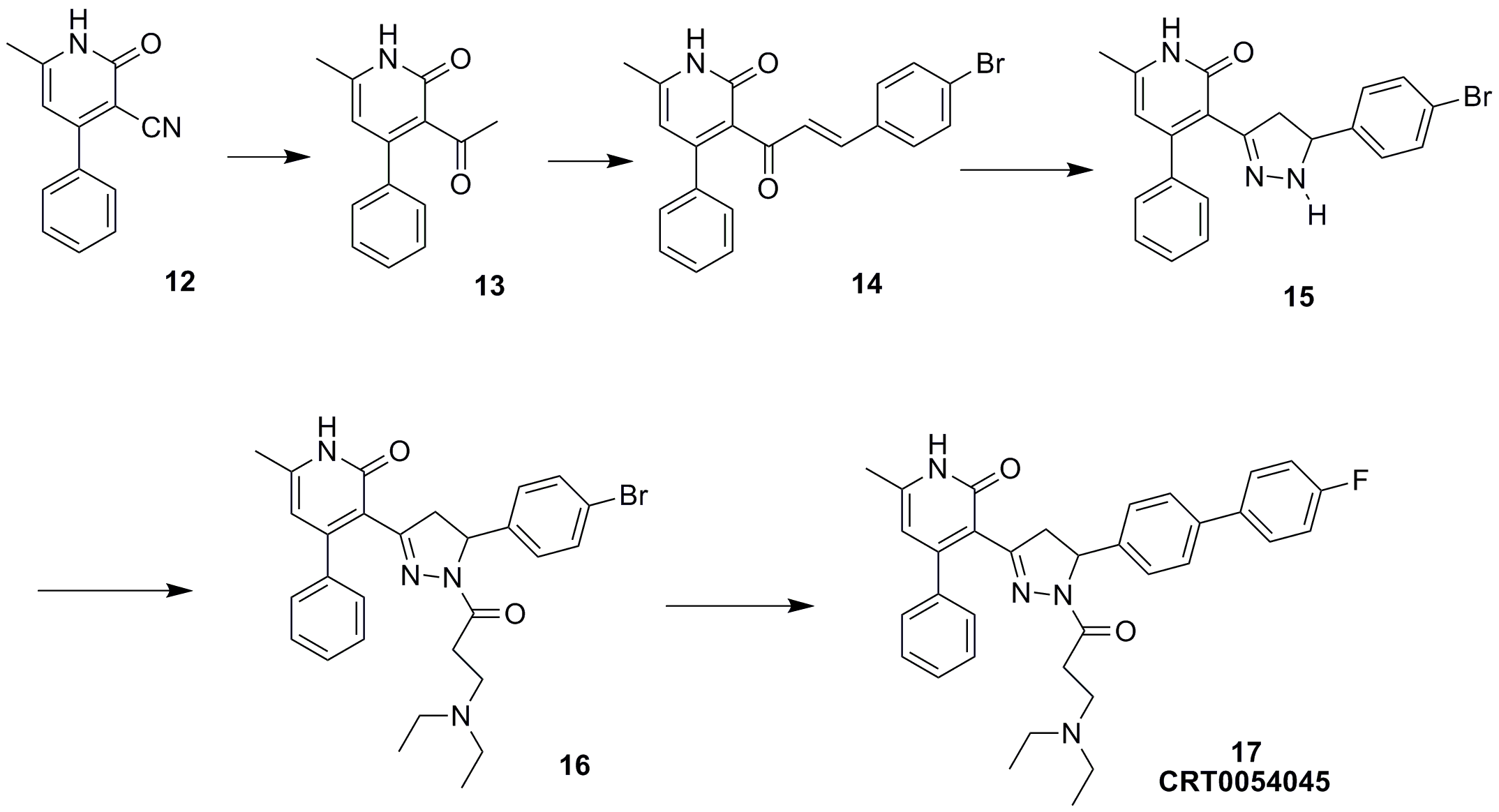


**Scheme 3**

### **6-Methyl-2-oxo-4-phenyl-1,2-dihydro-pryridine-3-carbonitrile (12):**

4-Phenyl-3-butene-2-one (25.0 g, 171 mmol) and cyanoacetamide (14.4 g, 171 mmol) were partially dissolved in anhydrous 1-butanol (150 mL). Piperidine (4.51 mL, 51.0 mmol) was added and the reaction mixture was heated up to 110°C under nitrogen for 24 hours. The reaction was left to cool and after one hour a precipitate formed which was filtered off to give 6-methyl-2-oxo-4-phenyl-1,2-dihydro-pryridine-3-carbonitrile as a pale yellow solid (**12**, 15.0 g, 36%). Analytical LCMS method 2: Rt 5.17 min; M-H = 209; ^1^H-NMR (d_6_-DMSO, 300MHz) δ: 2.30 (3H, s), 6.34 (1H, s), 7.51-7.60 (5H, m), 12.60 (1H, s).

### **3-Acetyl-6-methyl-4-phenyl-1H-pyridin-2-one (13):**

6-methyl-2-oxo-4-phenyl-1,2-dihydro-pryridine-3-carbonitrile (**12,** 2.10 g, 10.0 mmol) was dissolved under nitrogen in a mixture diethyl ether/THF (17 mL/ 13 mL) under nitrogen. The reaction was cooled down to 0°C and methyl magnesium iodide (3 M solution in THF, 50 mmol, 16.7 mL) was the added slowly to the mixture. The reaction was left to stir at room temperature overnight under nitrogen. The reaction was cooled down to 0°C and carefully quenched with water (30 mL) and then 3N hydrochloric acid (200 mL) was added. A clear yellow solution was obtained which was allowed to stay at room temperature over night. The precipitate formed was filtered off to give 3-acetyl-6-methyl-4-phenyl-1H-pyridin-2-one as a crystalline solid (**13**, 1.95 g, 86%). Analytical LCMS method 2: Rt 5.24 min; M+H = 228; ^1^H-NMR (CDCL_3_, 300MHz) δ: 2.32 (3H, s), 2.40 (3H, s), 5.85(1H, s), 7.50-7.30 (5H, m).

### **3-[(E)-3-(4-Bromo-phenyl)-acryloyl]-6-methyl-4-phenyl-1H-pyridin-2-one (14):**

To a solution of 3-[(E)-3-(4-chloro-phenyl)-acryloyl]-6-methyl-4-phenyl-1H-pyridin-2-one (**13,** 4 g, 17.6 mmol) in ethanol/water (1:1, 80ml) was added 4-bromobenzaldehyde (3.69 g, 21.1 mmol) and potassium hydroxide (2.9 g, 52.8 mmol). The reaction mixture was stirred at room temperature for 12 hours. The solvent was then evaporated *in vacuo* to give 3-[(*E*)-3-(4-bromo-phenyl)-acryloyl]-6-methyl-4-phenyl-1H-pyridin-2-one (**14,** 6.94 g, 100%) which was used without further purification. Analytical LCMS method 1, Rt 5.89 min, M+H = 394/396.

### **3-[5-(4-bromo-phenyl)-4,5-dihydro-1H-pyrazol-3-yl]-6-methyl-4-phenyl-1H-pyridin-2-one (15):**

To 3-[(*E*)-3-(4-bromo-phenyl)-acryloyl]-6-methyl-4-phenyl-1H-pyridin-2-one (**14,** 6.94 g, 17.6 mmol) in ethanol (45 mL) in microwave tubes was added hydrazine hydrate (2.74 mL, 88 mmol). The reaction mixture was heated up to 100°C during 5 min in a microwave. The solvent was evaporated and ethyl acetate (15 mL) was added to the residue. A yellow precipitate appeared which was filtered to give 3-[5-(4-bromo-phenyl)-4,5-dihydro-1H-pyrazol-3-yl]-6-methyl-4-phenyl-1H-pyridin-2-one as a bright yellow solid (**15**, 6.5 g, 90%). Analytical LCMS method 1, Rt 5.70 min, M+H = 408.

### **3-[5-(4-Bromo-phenyl)-1-(3-diethylamino-propionyl)-4,5-dihydro-1H-pyrazol-3-yl]-6-methyl-4-phenyl-1H-pyridin-2-one (16):**

3-[5-(4-bromo-phenyl)-4,5-dihydro-1H-pyrazol-3-yl]-6-methyl-4-phenyl-1H-pyridin-2-one (**15**, 6.5 g, 15.92 mmol), diisopropylethylamine (5.56ml, 31.8mmol), 3-(diethylamino)propionic acid hydrochloride (3.47 g, 19.1mmol) and O-(1H-Benzotriazol-1-yl)-N,N,N’,N’-tetramethyl-uronium hexafluorophosphate (7.26 g, 19.1mmol) were dissolved in dry dimethylacetamide (20mL) and left to stir overnight at room temperature. The solvent was then evaporated *in vacuo* and the residue purified by column chromatography (dichloromethane/methanol 99/1 with 0.5% NH_3_ to dichloromethane/methanol 90/10 with 0.5% NH_3_) to give 3-[5-(4-Bromo-phenyl)-1-(3-diethylamino-propionyl)-4,5-dihydro-1H-pyrazol-3-yl]-6-methyl-4-phenyl-1H-pyridin-2-one (**16,** 3.56 g, 42%). Analytical LCMS method 1, Rt 5.01 min, M+H = 535-537. ^1^H-NMR (CDCl_3_, 300MHz) δ: 1.19 (t, 3H), 2.32 (s, 3H), 2.53-3.07 (m, 9H), 3.54-3.64 (dd, 1H, J=11.7Hz, J’=18.3Hz), 5.30-5.36 (dd, 1H, J=11.7Hz, J’’=4.1Hz), 6.14 (s, 1H), 6.98 (d, 2H), 7.21-7.39 (m, 8H).

**3-[1-(3-Diethylamino-propionyl)-5-(4’-fluoro-biphenyl-4-yl)-4,5-dihydro-1H-pyrazol-3-yl]-6-methyl-4-phenyl-1H-pyridin-2-one (17) CRT0054045:**

To 4-fluorobenzeneboronic acid (7.8 mg, 0.056 mmol) in a microwave vial was added a solution of 3-[5-(4-bromo-phenyl)-1-(3-diethylamino-propionyl)-4,5-dihydro-1H-pyrazol-3-yl]-6-methyl-4-phenyl-1H-pyridin-2-one (**16**, 30 mg, 0.056 mmol) in dimethylacetamide (0.8 mL) followed by potassium phosphate (24 mg, 0.112mmol) in water (0.2 mL). Tetrakis(triphenylphosphine)palladium (3.2 mg, 0.003 mmol) was then added and the reaction mixture heated up to 150°C in a microwave for 10 min. The reaction mixture was then passed through a plug of silica and the silica washed with methanol. Solvents were removed and the compound was purified by preparative HPLC (method Prep HPLC 1) to give 3-[1-(3-diethylamino-propionyl)-5-(4’-fluoro-biphenyl-4-yl)-4,5-dihydro-1H-pyrazol-3-yl]-6-methyl-4-phenyl-1H-pyridin-2-one as the formate salt (**17**, 10mg, 33%). Analytical LCMS method 2, Rt 3.81 min, M+H = 551. ^1^H-NMR (CDCl_3_, 300MHz) δ: 1.21 (t, 3H), 2.38 (s, 3H), 2.71-3.21 (m, 9H), 3.69-3.79 (dd, 1H, J=11.8Hz, J’=18.4Hz), 5.35-5.41 (dd, J=11.8Hz, J’=4.5Hz), 6.21 (s, 1H), 7.08-7.51 (m, 13H), 8.23 (s, formic acid, 2H).


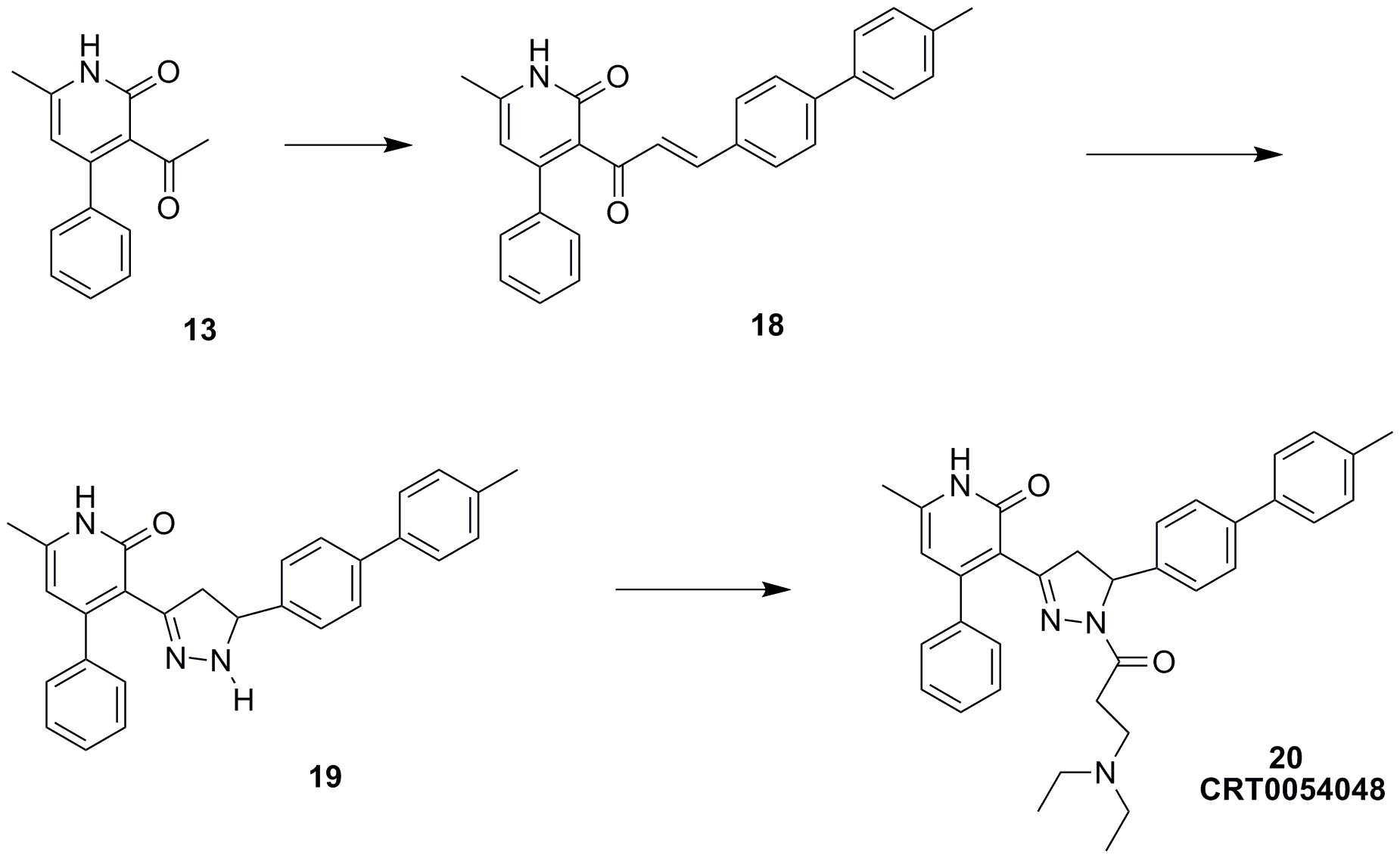


**Scheme 4**

**6-Methyl-3-[(*E*)-3-(4'-methyl-biphenyl-4-yl)-acryloyl]-4-phenyl-1H-pyridin-2-one (18):**

A solution of sodium hydroxide (1.7 g in 30 mL deionised water) was added to a solution of 3-acetyl-6-methyl-4-phenyl-1H-pyridin-2-one (**13**, 2.27 g, 10 mmol) and 4'-methyl-biphenyl-4-carbaldehyde (2.8 g, 14 mmol) in ethanol (30 mL). The reaction was left stirring at room temperature for 2 days. The solvent was then removed and the residue was purified by column chromatography (silica, ethyl acetate/cyclohexane; 1:4) to give 6-methyl-3-[(*E*)-3-(4'-methyl-biphenyl-4-yl)-acryloyl]-4-phenyl-1H-pyridin-2-one (**18**, 1.2 g 30%).Analytical LCMS method 1, Rt 6.25 min, M+H = 406.

**6-Methyl-3-[5-(4'-methyl-biphenyl-4-yl)-4,5-dihydro-1H-pyrazol-3-yl]-4-phenyl-1H-pyridin-2-one (19):**

6-Methyl-3-[(*E*)-3-(4'-methyl-biphenyl-4-yl)-acryloyl]-4-phenyl-1H-pyridin-2-one: (**18**, 0.2 g, 0.5 mmol) were dissolved in ethanol (5 mL). The vial was sealed and then hydrazine (0.1 mL, 3 mmol) was added and the vial was heated in a microwave for 10 minutes at 100°C. The solution was evaporated off *in vacuo* and 6-methyl-3-[5-(4'-methyl-biphenyl-4-yl)-4,5-dihydro-1H-pyrazol-3-yl]-4-phenyl-1H-pyridin-2-one (**19**, 0.21, 100%) was used without further purification. Analytical LCMS method 1, Rt 4.19 min, M+H = 420.

**3-[1-(3-Diethylamino-propionyl)-5-(4'-methyl-biphenyl-4-yl)-4,5-dihydro-1H-pyrazol-3-yl]-6-methyl-4-phenyl-1H-pyridin-2-one (20) CRT0054048:**

6-Methyl-3-[5-(4'-methyl-biphenyl-4-yl)-4,5-dihydro-1H-pyrazol-3-yl]-4-phenyl-1H-pyridin-2-one (**19**, 210mg, 5 mmol) was dissolved in dry dimethylformamide (5 mL) under nitrogen. Diisopropylethylamine (0.13 mL, 1 mmol) was then added followed by O-(1H-benzotriazol-1-yl)-N,N,N',N'-tetramethyl-uronium hexafluorophosphate (266 mg, 0.7 mmol) and 3-(diethylamino)propionic acid hydrochloride (130 mg, 0.7 mmol). After 3 hours the reaction was poured into water (50 mL) and extracted with ethyl acetate (3 x 30 mL). The organic phase was separated and dried over magnesium sulfate and evaporated *in vacuo*. The residue was then purified by column chromatography (silica, 2% methanol in dichloromethane) to give 3-[1-(3-diethylamino-propionyl)-5-(4'-methyl-biphenyl-4-yl)-4,5-dihydro-1H-pyrazol-3-yl]-6-methyl-4-phenyl-1H-pyridin-2-one (**20**, 95mg, 33%). Analytical LCMS method 1, Rt 5.06min, M+H = 547. 1H-NMR (CD_3_OD, 300 MHz) δ:  1.08 (t,6H, J=7.2Hz), 2.35 (s,3H), 2.36 (s,3H), 2.68 (m,6H), 3 (m,1H), 2.89 (m,3H), 5.43 (dd, 1H, J=3Hz, J=12 Hz), 6.26 (s,1H), 7.12 (d, 2H, J=9Hz), 7.24 (d, 2H, J=6Hz), 7.42 (m, 3H), 7.33 (m, 2H), 7.47 (m, 4H, J=6Hz).


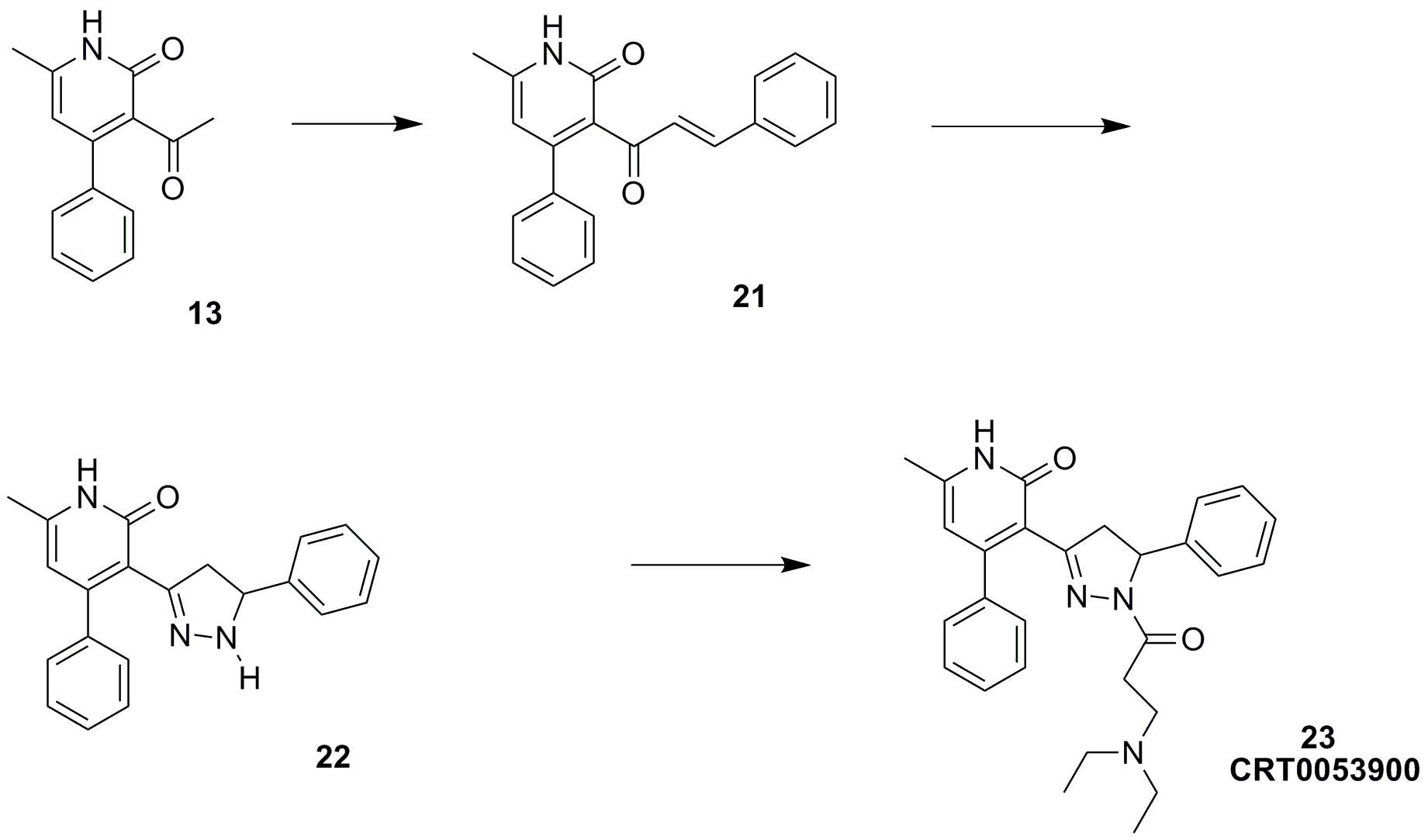


**Scheme 5**

### **6-Methyl-4-phenyl-3-[(E)-(3-phenyl-acryloyl)]-1H-pyridin-2-one (21):**

To a solution of 3-acetyl-6-methyl-4-phenyl-1H-pyridin-2-one (**13,** 1 g, 4.4 mmol) in ethanol/water (1:1, 50ml) was added benzaldehyde (0.67ml, 6.6mmol) and potassium hydroxide (0.49g, 8.8mmol). The reaction mixture was then stirred at room temperature for 12 hours. The solvent was evaporated and 30ml of water were added. The residue was extracted with ethyl acetate (2x40ml) and the organic were washed with brine and dried over magnesium sulphate. The solvent was evaporated and the residue purified by column chromatography (1:1, ethyl acetate/cyclohexane) to give 6-methyl-4-phenyl-3-[(*E*)-(3-phenyl-acryloyl)]-1H-pyridin-2-one (**21**, 0.6 g, 43%). Analytical LCMS method 1, Rt 5.59 min, M+H = 316. ^1^H-NMR (CDCl_3_, 300MHz) δ: 2.24 (s, 3H), 6.12 (s, 1H), 6.85 (s, 1H), 6.92(s, 1H), 7.27-7.39 (m, 10H), 12.11(s, 1H).

### **6-Methyl-4-phenyl-3-(5-phenyl-4,5-dihydro-1H-pyrazol-3-yl)1H-pyridin-2-one (22):**

### To 6-methyl-4-phenyl-3-[(E)-(3-phenyl-acryloyl)]-1H-pyridin-2-one (**21,** 0.5 g, 1.6 mmol) in ethanol (10ml) in a microwave tube was added hydrazine hydrate (0.24ml, 8mmol). The reaction mixture was heated up to 100°C during 5 min in a microwave. The solvent was then evaporated and 6-methyl-4-phenyl-3-(5-phenyl-4,5-dihydro-1H-pyrazol-3-yl)1H-pyridin-2-one (**22**, 0.47 g, 89%) was used without further purification. LCMS method 1, Rt 5.36 min and 5.49 min, M+H = 330. ^1^H-NMR (CDCl_3_) δ: 2.25 (s, 3H), 2.66-2.75 (dd, 1H, J=10.8Hz, J'=16.5Hz), 3.28-3.37 (dd, 1H, J=10.8Hz, J'=16.5Hz), 4.67 (m, 1H), 6.07 (s, 1H), 7.01 (s,1H), 7.25-7.44 (m, 10H).

**3-[1-(3-Diethylamino-propionyl)-5-phenyl-4,5-dihydro-1H-pyrazol-3-yl]-6-methyl-4-phenyl-1H-pyridin-2-one (23) CRT0053900:**

6-Methyl-4-phenyl-3-(5-phenyl-4,5-dihydro-1H-pyrazol-3-yl)1H-pyridin-2-one (**22**, 50mg, 0.15 mmol), diisopropylethylamine (53µl, 0.3mmol), 3-(diethylamino)propionic acid hydrochloride (33mg, 0.18mmol) and O-(1H-benzotriazol-1-yl)-N,N,N',N'-tetramethyl-uronium hexafluorophosphate (69mg, 0.18mmol) were taken up in dry dimethylacetamide (1ml). The reaction was left to stir at room temperature overnight. The solvent was evaporated *in vacuo* and the residue was taken forward for purification. The desired product was purified by preparative HPLC (Prep HPLC method 3) to give 3-[1-(3-diethylamino-propionyl)-5-phenyl-4,5-dihydro-1H-pyrazol-3-yl]-6-methyl-4-phenyl-1H-pyridin-2-one as a yellow oil (**23**, 28.9 mg, 42%). Analytical LCMS method 1, Rt 4.76 min, M+H = 457. ^1^H-NMR (CDCl_3_, 300MHz) δ: 1.17-1.27 (dt, 6H), 2.51 (s, 3H), 2.75-3.10 (m, 7H), 3.28 (m, 4H), 3.58-3.68 (dd, 1H, J=12.0Hz, J'=18.6Hz), 5.31-5.36 (dd, 1H, J=11.7Hz, J'=4.5Hz), 6.52 (s, 1H), 6.99-7.53 (m, 10H).


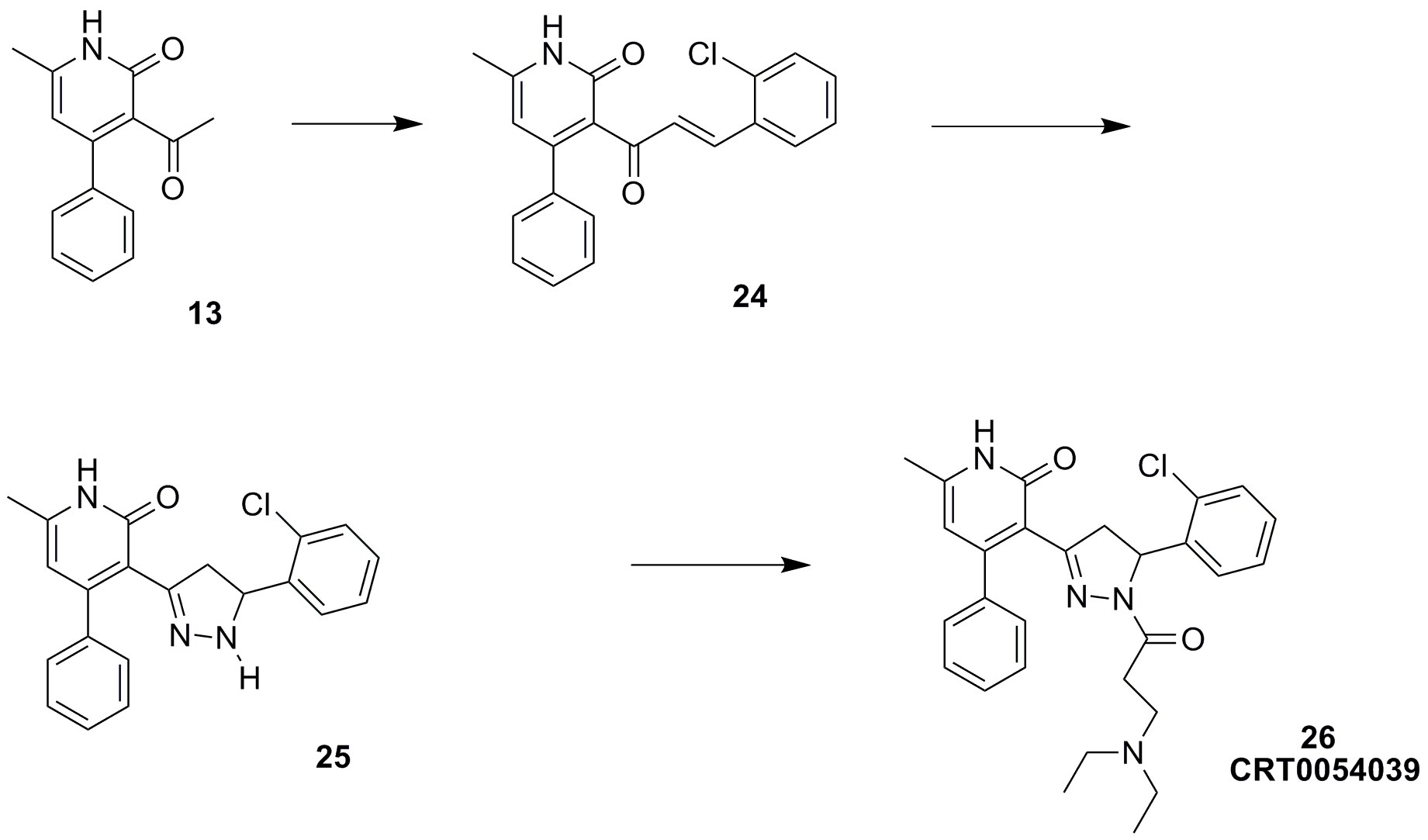


**Scheme 6**

### **3-[(E)-3-(2-Chloro-phenyl)-acryloyl]-6-methyl-4-phenyl-1H-pyridin-2-one (24):**

To a solution of 3-[(*E*)-3-(4-chloro-phenyl)-acryloyl]-6-methyl-4-phenyl-1H-pyridin-2-one (**13**, 0.5 g, 2.2 mmol) in ethanol/water (1:1, 20ml) was added 2-chlorobenzaldehyde (0.37, 3.3mmol) and potassium hydroxide (0.37g, 4.4mmol). The reaction mixture was stirred at room temperature for 12 hours and the solvent was evaporated. 3-[(E)-3-(2-chloro-phenyl)-acryloyl]-6-methyl-4-phenyl-1H-pyridin-2-one (**24**, 0.77g, 100%) was used without further purification. Analytical LCMS method 1, Rt 5.77 min, M+H = 350.

### **3-[5-(2-Chloro-phenyl)-4,5-dihydro-1H-pyrazol-3-yl]-6-methyl-4-phenyl-1H-pyridin-2-one (25):**

To 3-[(*E*)-3-(2-Chloro-phenyl)-acryloyl]-6-methyl-4-phenyl-1H-pyridin-2-one (**24**, 0.77 g, 2.2 mmol) in ethanol (10ml) in a microwave tube was added hydrazine hydrate (0.34ml, 2.64mmol). The reaction mixture was heated up to 100°C during 5 min in a microwave. The solvent was evaporated *in vacuo* and the residue, 3-[5-(2-Chloro-phenyl)-4,5-dihydro-1H-pyrazol-3-yl]-6-methyl-4-phenyl-1H-pyridin-2-one (**25,** 0.80 g, 100%) was used without further purification. Analytical LCMS method 1, Rt 5.62 min, M+H = 364.

### **3-[5-(2-Chloro-phenyl)-1-(3-diethylamino-propionyl)-4,5-dihydro-1H-pyrazol-3-yl]-6-methyl-4-phenyl-1H-pyridin-2-one (26) CRT0054039:**

3-[5-(2-Chloro-phenyl)-4,5-dihydro-1H-pyrazol-3-yl]-6-methyl-4-phenyl-1H-pyridin-2-one (**25**, 50mg, 0.137mmol)), diisopropylethylamine (21mg, 0.16mmol), 3-(diethylamino)propionic acid hydrochloride (55mg, 0.3mmol) and O-(1H-benzotriazol-1-yl)-N,N,N',N'-tetramethyl-uronium hexafluorophosphate (62mg, 0.16mmol) were taken up in dry dimethylacetamide (1.5ml). The reaction was left to stir at room temperature overnight. The solvent was evaporated under vacuum and compounds were taken forward for purification by preparative HPLC. 3-[5-(2-Chloro-phenyl)-1-(3-diethylamino-propionyl)-4,5-dihydro-1H-pyrazol-3-yl]-6-methyl-4-phenyl-1H-pyridin-2-one was purified using Prep HPLC Method 3 to give of a yellow oil (**26**, 10.9mg, 14%). Analytical LCMS method 1, Rt 4.82min, M+H = 491.5. ^1^H-NMR (CDCl_3_, 300MHz) δ: 1.25-1.30 (t, 6H), 2.47 (s, 3H), 2.71-3.33 (m, 9H), 3.68-3.78 (dd, 1H, J= 18.6Hz, J'=11.7Hz), 5.67-5.73 (dd, 1H, J'=11.7Hz, J''=4.8Hz), 6.43 (s, 1H), 6.74-7.47 (m, 9H)., 11.47 (s, 1H).

**References**

5. Davenport EL, Moore HE, Dunlop AS, Sharp SY, Workman P, et al. (2007) Heat shock protein inhibition is associated with activation of the unfolded protein response pathway in myeloma plasma cells. Blood 110: 2641-2649.
